# A Role for Chromatin Remodeling in Cohesin Loading onto Chromosomes

**DOI:** 10.1016/j.molcel.2019.02.027

**Published:** 2019-05-16

**Authors:** Sofía Muñoz, Masashi Minamino, Corella S. Casas-Delucchi, Harshil Patel, Frank Uhlmann

**Affiliations:** 1 Chromosome Segregation Laboratory, The Francis Crick Institute, 1 Midland Road, London NW1 1AT, UK; 2 Chromosome Replication Laboratory, The Francis Crick Institute, 1 Midland Road, London NW1 1AT, UK; 3 Bioinformatics and Biostatistics Science Technology Platform, The Francis Crick Institute, 1 Midland Road, London NW1 1AT, UK

**Keywords:** cohesin, cohesin loader, Scc2-Scc4, sister chromatid cohesion, RSC, chromatin remodeling, *Saccharomyces cerevisiae*

## Abstract

Cohesin is a conserved, ring-shaped protein complex that topologically embraces DNA. Its central role in genome organization includes functions in sister chromatid cohesion, DNA repair, and transcriptional regulation. Cohesin loading onto chromosomes requires the Scc2-Scc4 cohesin loader, whose presence on chromatin in budding yeast depends on the RSC chromatin remodeling complex. Here we reveal a dual role of RSC in cohesin loading. RSC acts as a chromatin receptor that recruits Scc2-Scc4 by a direct protein interaction independent of chromatin remodeling. In addition, chromatin remodeling is required to generate a nucleosome-free region that is the substrate for cohesin loading. An engineered cohesin loading module can be created by fusing the Scc2 C terminus to RSC or to other chromatin remodelers, but not to unrelated DNA binding proteins. These observations demonstrate the importance of nucleosome-free DNA for cohesin loading and provide insight into how cohesin accesses DNA during its varied chromosomal activities.

## Highlights

• Nucleosomes inhibit cohesin loading
• Chromatin remodeling by RSC promotes cohesin loading
• RSC recruits Scc2-Scc4 to chromatin independent of remodeling
• Linking Scc2-C to various chromatin remodelers recreates cohesin loading

## Graphical Abstract

**Figure:**
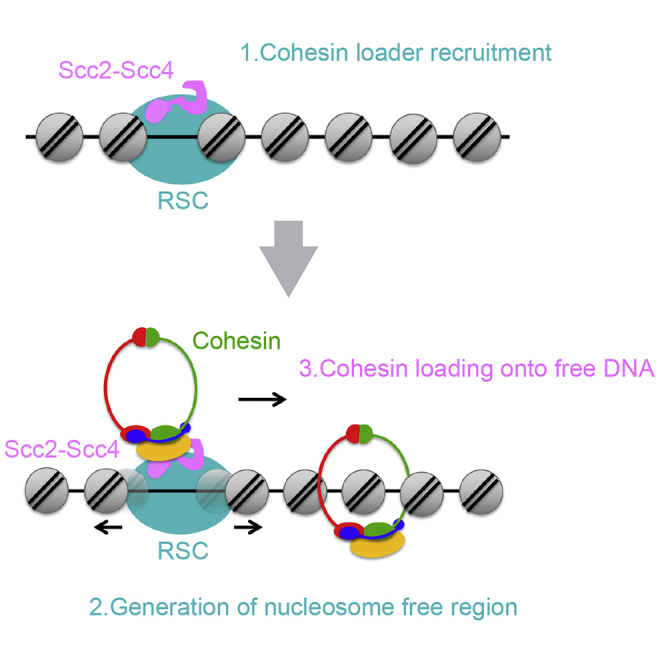

## Teaser

Despite our increasing understanding of cohesin, how this essential protein complex accesses chromosomes is incompletely understood. Muñoz et al. provide insight into this by revealing that a molecular machine that mobilizes nucleosomes assists with cohesin loading and defines the DNA entry points for cohesin in the context of chromatin.

## Introduction

Following DNA replication, sister chromatids are held together in a process known as sister chromatin cohesion. This is essential for faithful chromosome segregation during cell divisions. Sister chromatid cohesion is achieved by cohesin, a ring-shaped protein complex that topologically entraps both sister chromatids (Nasmyth and Haering, 2009, Peters and Nishiyama, 2012, Uhlmann, 2016). The cohesin complex consists of two structural maintenance of chromosomes (SMC) proteins, Smc1 and Smc3, characterized by long flexible coiled coils that dimerize at a hinge domain. At their far end lie globular ATPase heads that engage in the presence of ATP and are bridged by a kleisin subunit, Scc1, to complete the ring. Additional HEAT repeats containing subunits, Scc3 and Pds5, associate with the kleisin as well as a substoichiometric regulator, Wapl. Apart from its prominent role in sister chromatid cohesion, cohesin takes part in many other chromosomal processes, including DNA repair and organization of the genome into chromatin loops (Dorsett and Ström, 2012, van Ruiten and Rowland, 2018).

Cohesin association with chromatin depends on a second protein complex comprised of the Scc2 and Scc4 subunits (Ciosk et al., 2000). This cohesin loader complex makes multiple contacts with both cohesin and DNA that facilitate topological cohesin loading *in vitro* (Murayama and Uhlmann, 2014). In the presence of DNA, the cohesin loader stimulates cohesin’s ATPase, which forms part of the DNA loading reaction. Structural and biochemical studies have shown that the cohesin loader consists of two functional modules (Chao et al., 2015, Hinshaw et al., 2015, Kikuchi et al., 2016, Takahashi et al., 2008). DNA and cohesin interactions reside in a C-terminal portion of Scc2 (Scc2C) that largely consists of HEAT repeats, resembling cohesin’s two other HEAT subunits. Scc2C is sufficient to catalyze cohesin loading onto naked DNA *in vitro* but fails to load cohesin onto chromosomes *in vivo*. Scc4 forms a tetratricopeptide repeat superhelix that wraps around the Scc2 N terminus (Scc4-Scc2N). This module recruits the cohesin loader to chromatin *in vivo* (Chao et al., 2015, Hinshaw et al., 2015). Scc4-Scc2N has no intrinsic affinity for DNA, suggesting that its interaction with chromatin occurs via a protein receptor.

*In vitro*, Scc2-Scc4 loads cohesin in a DNA sequence-independent manner (Murayama and Uhlmann, 2014), whereas *in vivo* cohesin is loaded at specific chromosomal locations, at centromeres and promoters of certain highly transcribed genes (Kagey et al., 2010, Lopez-Serra et al., 2014, Petela et al., 2018, Zuin et al., 2014). From there, cohesin slides, pushed by the transcription machinery, to its final chromosomal destinations in pericentromeric regions and at sites of convergent transcriptional termination. Along human chromosomes, the CCCTC-binding factor (CTCF) forms alternative cohesin retention sites (Busslinger et al., 2017, Davidson et al., 2016, Ocampo-Hafalla et al., 2016). The chromatin features that define cohesin loading sites are incompletely understood. At budding yeast centromeres, an interaction between the inner kinetochore protein Ctf19 and a conserved surface patch on Scc4 contributes to cohesin loader recruitment. This depends on Ctf19 N-terminal phosphorylation by the Dbf4-dependent kinase (DDK) (Hinshaw et al., 2017). This pathway enhances, but is not essential for, cohesin loading at centromeres. DDK also mediates cohesin loader recruitment to pre-replicative complexes to achieve cohesin loading onto transcriptionally inactive chromosomes in *Xenopus* cell-free extracts (Takahashi et al., 2008). Despite this insight, the nature of the essential pathway that loads cohesin in the chromatin context of transcriptionally active chromosome arms remains incompletely understood.

Comparison of Scc2-Scc4 binding sites with those of other chromatin factors in budding yeast revealed an overlap with the remodels the structure of chromatin’ (RSC) chromatin remodeling complex (Lopez-Serra et al., 2014). RSC is a yeast ortholog of the human BAF and PBAF complexes, members of the conserved SWI/SNF family of ATP-dependent chromatin remodelers. They are large multisubunit protein complexes that push DNA along the histone octamer, leading to nucleosome sliding or eviction. Either outcome opens up chromatin and renders it accessible to factors involved in various aspects of DNA metabolism, including transcription and DNA repair (Clapier et al., 2017, Lorch and Kornberg, 2017). RSC maintains broad nucleosome-free regions at promoters where the cohesin loader is found (Lopez-Serra et al., 2014). However, it is not yet known how RSC recruits the cohesin loader or whether chromatin remodeling forms part of the cohesin loading reaction.

Here we investigate the role of RSC in cohesin loading onto chromosomes. This reveals a dual role for this chromatin remodeler. First, RSC serves as the chromatin receptor of the cohesin loader by engaging in a direct protein interaction with the Scc2 and Scc4 subunits. This recruitment role does not require chromatin remodeling. In addition, chromatin remodeling provides nucleosome-free DNA, which is the required substrate for cohesin loading. The cohesin loading function of Scc2C can be reassigned from RSC to other chromatin remodelers but not to other DNA binding proteins. This establishes a close relationship between chromatin remodeling and cohesin loading onto chromosomes, describing the entry point by which cohesin accesses DNA in the context of chromatin.

## Results

### The RSC ATPase Is Required for Cohesin Loading

**Figure 1:**
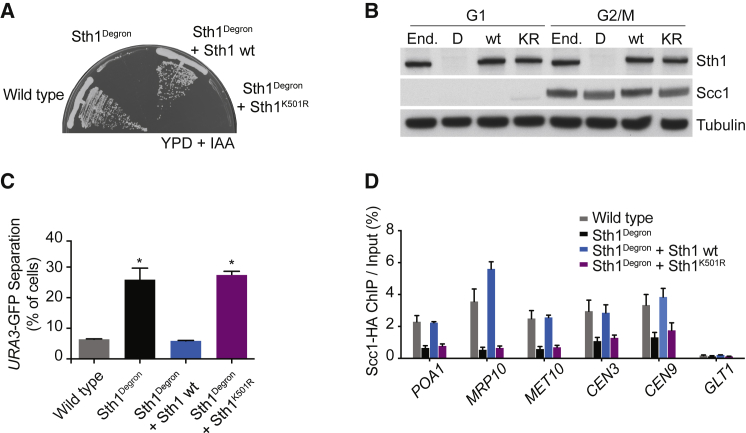
Cohesin Loading Requires RSC Catalytic Activity (A) The ATPase mutant Sth1 fails to restore cell viability following Sth1 depletion. Wild-type Sth1^Degron^ cells and Sth1^Degron^ cells expressing wild-type Sth1 or Sth1^K501R^ were streaked onto rich yeast extract peptone dextrose (YPD) medium containing methionine to repress Sth1^Degron^ expression and indole-3-acetic acid (IAA) to promote its degradation. (B) Ectopic Sth1 and Sth1^K501R^ are expressed at similar levels as endogenous Sth1. Cells of the indicated genotypes were synchronized in G1, endogenous Sth1 was depleted, and cells were released into nocodazole-imposed mitotic arrest. Levels of Sth1 and of the cohesin subunit Scc1 were monitored by immunoblot. Tubulin served as a loading control. End., endogenous Sth1; D, Sth1^Degron^; wild-type (wt), Sth1^Degron^ + wild-type Sth1; KR, Sth1^Degron^ + Sth1^K501R^. (C) RSC catalytic activity is required for sister chromatid cohesion; as in (B), but sister chromatid cohesion at the GFP-marked *URA3* locus was scored. Means and SEM of three independent experiments are shown. Sth1^Degron^ and Sth1^Degron^ + Sth1^K501R^, p < 0.01; Sth1^Degron^ + Sth1 wt, p not significant; Student’s t test compared with the wild-type strain. (D) RSC catalytic activity promotes cohesin loading; as in (B), but Scc1 levels at three chromosome arm cohesin binding sites *(POA1*, *MRP10*, and *MET10*), two centromeres (*CEN3* and *CEN9*), and a negative control site (*GLT1*) were measured by ChIP, followed by real-time qPCR. Means and SEM of three independent experiments are shown. Sth1^Degron^ and Sth1^Degron^ + Sth1^K501R^, p < 0.01; Sth1^Degron^ + Sth1 wt, p not significant; two-way ANOVA test compared with the wild-type strain. See also Figure S1 for a schematic of cell synchronization and cell cycle progression analysis by fluorescence-activated cell sorting (FACS) analysis of DNA content as well as Figure S2 for additional ChIP microarray and quantitative analyses of the cohesin distribution.

RSC chromatin remodeler inactivation leads to loss of cohesin from chromosomes and defective sister chromatid cohesion as well as reduced Scc2-Scc4 levels at cohesin loading sites (Baetz et al., 2004, Huang et al., 2004, Lopez-Serra et al., 2014). To investigate whether chromatin remodeling by RSC is required for these functions, we conditionally depleted Sth1, its essential catalytic RecA-type ATPase subunit. We achieved this by replacing the *STH1* promoter with the methionine-repressible *MET3* promoter, combined with Sth1 fusion to an auxin-inducible degron tag (Nishimura et al., 2009). In this background, we introduced an additional copy of either the wild-type *STH1* gene or *sth1*^*K501R*^ carrying an amino acid substitution within the ATP binding motif that abrogates ATP hydrolysis (Du et al., 1998). As expected, Sth1 depletion resulted in lethality. Cell growth could be rescued by expression of wild-type Sth1 but not Sth1^K501R^ (Figure 1A).

For the following experiments, we depleted Sth1 in G1-arrested cells and released cells to progress through one cell cycle until they were arrested again in G2/M by nocodazole treatment (Figures S1A and S1B). The absence of functional Sth1 reproducibly delayed DNA replication by around 15 min. Samples for analysis were taken after all cells had completed S phase. Western blotting confirmed efficient Sth1 depletion as well as ectopic expression levels of wild-type Sth1 or Sth1^K501R^, similar to endogenous Sth1 (Figure 1B). To evaluate sister chromatid cohesion, we monitored the GFP-marked *URA3* locus. Consistent with previous studies (Baetz et al., 2004, Huang et al., 2004, Lopez-Serra et al., 2014), Sth1 depletion resulted in defective sister chromatid cohesion. Cohesion was restored by expression of wild-type Sth1. In contrast, Sth1^K501R^ expression did not improve sister chromatid cohesion (Figure 1C).

We next asked whether Sth1^K501R^ failed to rescue sister chromatid cohesion because Sth1 catalytic activity is required for cohesin loading onto chromosomes. The cohesin subunit Scc1 is cleaved in anaphase and must be newly synthesized before cells enter the next round of DNA replication. We therefore first asked whether Scc1 expression is affected by Sth1 depletion. The kinetics and levels of Scc1 accumulation were indistinguishable between wild-type and Sth1-depleted cells (Figures 1B and S1C). Despite cohesin’s presence, quantitative chromatin immunoprecipitation (ChIP) analysis revealed markedly reduced cohesin levels following Sth1 depletion, both at centromeres and chromosome arms (Figure 1D). We note that low cohesin levels, detectable above background, persist following Sth1 depletion. Its chromosomal distribution remained largely unaltered, indicative of a global cohesin reduction rather than redistribution because of transcriptional changes or an altered chromatin landscape (Figure S2). Full chromosomal cohesin was restored by expression of wild-type Sth1 but not Sth1^K501R^ (Figure 1D). As an independent indicator for chromosome association, we observed the electrophoretic mobility of cohesin’s Scc1 subunit. Polo kinase-dependent Scc1 phosphorylation preferentially targets chromosome-bound cohesin (Hornig and Uhlmann, 2004). The corresponding mobility shift was reduced in Sth1-depleted cells and restored by wild-type Sth1 but not Sth1^K501R^ (Figure 1B). This supports the notion that efficient cohesin loading onto chromosomes depends on an active RSC ATPase.

### Cohesin Loader Recruitment Is Independent of Chromatin Remodeling

**Figure 2:**
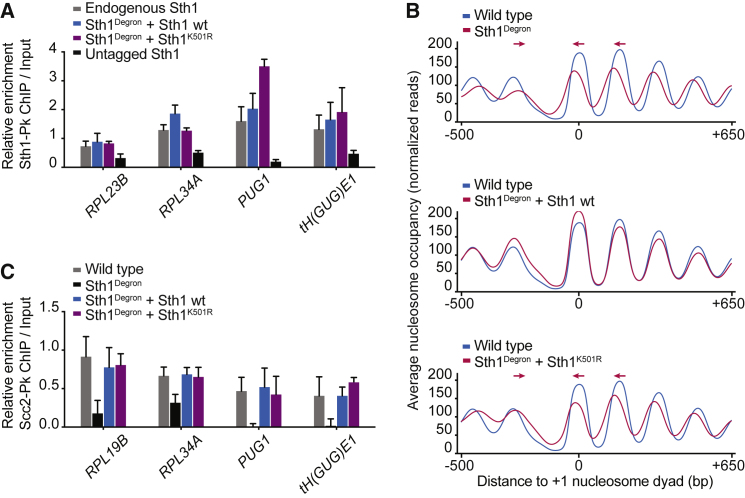
RSC Recruits the Cohesin Loader Independently of Its ATPase (A) Sth1^K501R^ is present on chromatin. Sth1 ChIP was performed in mitotic arrest following Sth1 depletion, as in Figure 1. Binding to three gene promoters (*RPL23B*, *RPL34A*, and *PUG1*) and a tRNA gene (*tH(GUG)E1*) was measured by real-time qPCR, normalized to a negative control site (*CIN8*). Means and SEM of three independent experiments are shown. Sth1^Degron^ + Sth1 wt and Sth1^Degron^ + Sth1^K501R^, p not significant; untagged Sth1, p < 0.01; two-way ANOVA test compared with the endogenous Sth1 strain. (B) The Sth1 ATPase is required for chromatin remodeling. Shown are average nucleosome occupancy profiles at 4,264 genes aligned to the +1 nucleosome midpoint, comparing wild-type with Sth1^Degron^ cells following Sth1 depletion and with Sth1^Degron^ cells expressing either Sth1 or Sth1^K501R^. (C) Cohesin loader recruitment by Sth1^K501R^. Scc2 levels were measured by ChIP, followed by real-time qPCR as in (A). Means and SEM of three independent experiments are shown. Sth1^Degron^, p < 0.01; Sth1^Degron^ + Sth1 wild-type and Sth1^Degron^ + Sth1^K501R^, p not significant; two-way ANOVA test compared with the wild-type strain.

To understand how the RSC ATPase facilitates cohesin loading, we first addressed whether chromatin association of the RSC complex depends on its ATPase. We compared wild-type Sth1 and Sth1^K501R^ occupancy at four previously characterized RSC binding sites (Lopez-Serra et al., 2014, Ng et al., 2002). Ectopic wild-type Sth1 associated with these sites at levels comparable with endogenous Sth1. The same was true for Sth1^K501R^ (Figure 2A), suggesting that remodeling activity is not required for RSC chromatin recruitment.

RSC establishes a nucleosome-depleted region within budding yeast promoters, upstream of a well-positioned +1 nucleosome that contains the transcription start site. Sth1 depletion leads to narrowing of this region, as seen by micrococcal nuclease digestion of chromatin followed by high-throughput sequencing. This is accompanied by a pronounced inward shift of the flanking nucleosomes in the gene body (Figure 2B; Hartley and Madhani, 2009, Kubik et al., 2018). Expression of wild-type Sth1 restored the nucleosome-depleted region as well as the original nucleosome positioning within gene bodies. In contrast, Sth1^K501R^ was unable to change nucleosome positioning over that seen following Sth1 depletion (Figure 2B). This confirms that RSC’s effect on promoter nucleosome positioning is principally mediated by its remodeling activity.

We then tested whether Scc2-Scc4 recruitment to cohesin loading sites depends on the nucleosome landscape. We measured Scc2 levels at the four cohesin loader binding sites, co-occupied by RSC, by quantitative ChIP. This analysis confirmed that cohesin loader recruitment depends on the RSC complex (Lopez-Serra et al., 2014). Scc2 occupancy was greatly reduced following Sth1 depletion. Scc2 levels were restored by expression of wild-type Sth1 and, to a similar extent, by expression of Sth1^K501R^ (Figure 2C). Thus, RSC recruits the cohesin loader independent of chromatin remodeling, possibly via a direct protein interaction. We noticed that Scc2 loss following Sth1 depletion was less pronounced at two ribosomal gene promoters (*RPL19B* and *RPL34A*) compared with the *PUG1* promoter and the *tH(GUC)E1* tRNA gene. Maybe a weaker alternative Scc2-Scc4 receptor exists at ribosomal protein gene promoters. In conclusion, RSC recruits the cohesin loader independent of its ATPase, but the ATPase is required to facilitate cohesin loading.

### Nucleosomes Interfere with Cohesin Loading *In Vitro*

**Figure 3:**
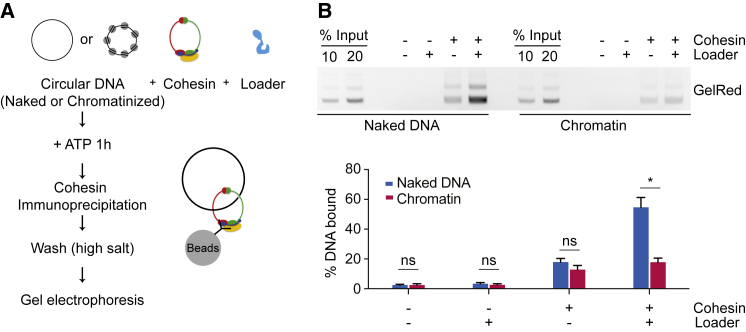
Nucleosomes Inhibit *In Vitro* Cohesin Loading (A) Schematic of the *in vitro* cohesin loading assay. (B) Gel image showing DNA retrieved by cohesin after a loading reaction in the presence or absence of cohesin and/or the cohesin loader, using either free plasmid DNA or its chromatinized derivative as a template. The graph shows means and SEM of three independent experiments. ^∗^p < 0.01; ns, not significant; Student’s t test. See also Figure S3 for the purification of budding yeast cohesin and its cohesin loader, a demonstration of topological *in vitro* cohesin loading onto DNA, and a micrococcal nuclease control for chromatin assembly.

To test whether nucleosome-free DNA is the required substrate for cohesin loading, we employed an *in vitro* assay that recapitulates cohesin loading onto DNA with purified proteins. We previously reconstituted topological DNA binding by the cohesin ring using fission yeast proteins (Murayama and Uhlmann, 2014). We now purified budding yeast cohesin and its loader for use in a similar assay (Figures 3A and S3A; Minamino et al., 2018). Following incubation of cohesin with circular DNA in the presence of the cohesin loader and ATP, cohesin was retrieved from the reaction by immunoprecipitation. The precipitate was washed, and the recovered DNA was analyzed by gel electrophoresis. Topological DNA capture was confirmed by loss of entrapment following DNA linearization (Figure S3B).

We now converted the template plasmid used for the loading reaction into evenly spaced nucleosomes using recombinant yeast histones, the yeast histone chaperone Nap1, and the yeast ISW1a chromatin remodeler (Kurat et al., 2017). Successful nucleosome assembly was confirmed following micrococcal nuclease digestion (Figure S3C). We then used this chromatinized template next to the nucleosome-free plasmid as substrate in the cohesin loading reaction. After incubation with cohesin and ATP, a small amount of either free or chromatinized DNA was recovered. Addition of the cohesin loader markedly stimulated cohesin loading onto nucleosome-free DNA but not onto chromatinized DNA (Figure 3B). This suggests that nucleosomes interfere with cohesin loading and that the Scc2-Scc4 complex requires nucleosome-free DNA as a substrate for cohesin loading. *In vivo*, all chromosomal cohesin loading depends on the loader (Ciosk et al., 2000, Watrin et al., 2006). This suggests that chromatin remodeling to access DNA forms an integral part of the cohesin loading reaction.

### RSC Directly Interacts with Cohesin and the Cohesin Loader

**Figure 4:**
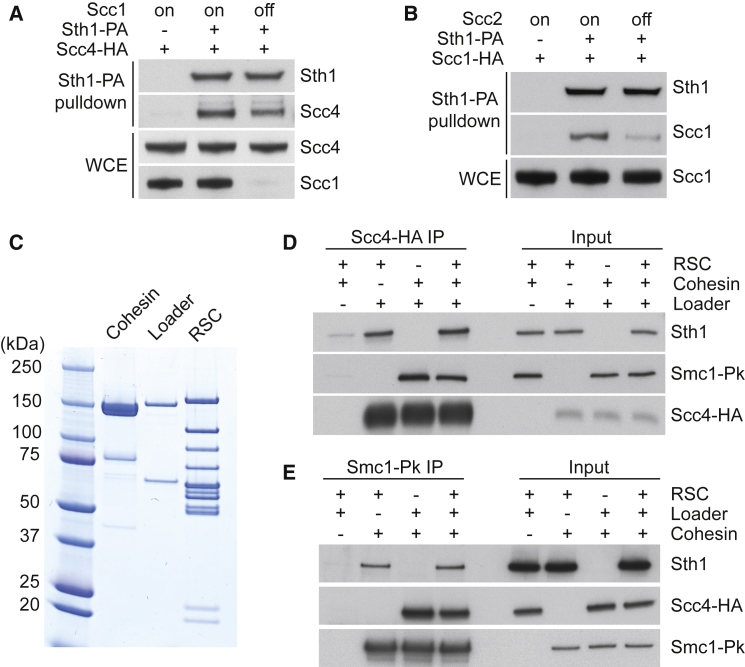
RSC, Cohesin, and the Cohesin Loader Interact Directly (A) Interaction between Sth1 and Scc4. Cells were synchronized in G1 and released into nocodazole-imposed mitotic arrest. Scc1 was depleted in one culture in G1 by methionine-induced promoter repression. Cell extracts were prepared, and protein A-tagged Sth1 was precipitated. Co-precipitation of Scc4 was analyzed by immunoblotting. (B) Interaction between Sth1 and cohesin; as in (A), but co-precipitation of the cohesin subunit Scc1 with protein A-tagged Sth1 was evaluated by immunoblotting. Scc2 was depleted in one culture by combination of promoter repression and an auxin-inducible degron. (C) Coomassie-stained gel showing purified cohesin, cohesin loader, and RSC chromatin remodeling complexes. (D) RSC and the cohesin loader interact directly. Equimolar amounts of RSC, cohesin, and cohesin loader were mixed as indicated. The cohesin loader was immunoprecipitated by its hemagglutinin (HA) epitope-tagged Scc4 subunit, and the co-precipitation of RSC or cohesin was analyzed by immunoblotting. (E) RSC and cohesin interact directly. Interaction analyses were performed as in (D), but cohesin was immunoprecipitated by its Pk epitope-tagged Smc1 subunit. See also Figure S4 for a comparison of the Sth1-Scc4 interaction between G1 and mitotic cells, an interaction assay using Sth1^K501R^, and an analysis of the cohesin-cohesin loader interaction.

RSC recruits the cohesin loader independently of chromatin remodeling. To test whether this involves a protein interaction between RSC and the cohesin loader, we fused a protein A tag to Sth1 and performed protein A pull-down experiments. We synchronized cells by α-factor pheromone block and release and subsequently arrested them in G2/M by nocodazole treatment, a cell cycle stage when both cohesin and the cohesin loader are present. Sth1 pull-down resulted in clearly detectable co-precipitation of Scc4 (Figure 4A). To test whether this interaction depended on cohesin, we included an experiment in which Scc1 expression was repressed under control of the methionine-repressible *MET3* promoter during and following the α-factor block. In the absence of Scc1, Sth1 still co-precipitated Scc4, albeit with somewhat reduced efficiency. We reached a similar conclusion when we compared Scc4-Sth1 co-precipitation in G2/M cells with cells arrested in G1, when little cohesin is present (Figure S4A). Consistent with the expectation that the RSC-cohesin loader interaction is independent of chromatin remodeling, Sth1^K501R^ also co-precipitated Scc4 (Figure S4B).

Because cohesin appeared to stabilize the RSC-cohesin loader interaction, RSC might also interact with cohesin. Indeed, a similar experimental approach revealed co-precipitation of the cohesin subunit Scc1 with Sth1 (Figure 4B). This interaction was reduced, but remained detectable, when Scc2 was depleted. To complete the interaction analysis, we confirmed the previously known interaction between cohesin and the cohesin loader and found that it is unaffected by the presence or absence of Sth1 (Figure S4C). Taken together, these findings establish three way interactions between RSC, cohesin, and the cohesin loader.

To decide whether RSC directly interacts with the cohesin loader and cohesin or whether the interactions are mediated by additional binding partners, we purified the RSC complex from budding yeast (Figure 4C; Wittmeyer et al., 2004). Pull-down of the purified cohesin loader led to co-purification of RSC both in the presence or absence of cohesin (Figure 4D). Similarly, cohesin pull-down demonstrated a direct interaction with RSC in both the presence or absence of the cohesin loader (Figure 4E). RSC, the cohesin loader, and cohesin were purified in the presence of benzonase, and the interaction analyses were additionally supplemented with benzonase, suggesting that direct protein interactions take place between RSC, the cohesin loader, and cohesin.

### Both Scc4 and Scc2 Contribute to the RSC Interaction

**Figure 5:**
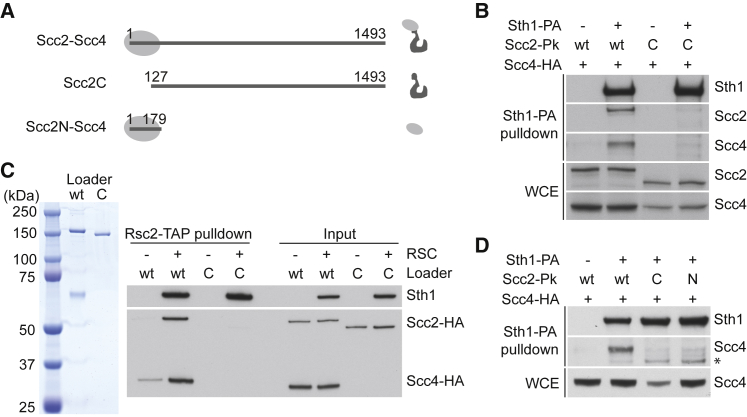
Both Scc4 and Scc2 Contribute to the RSC Interaction (A) Schematic of cohesin loader modules used in this experiment. (B) Scc2C shows reduced interaction with RSC. Cell extracts from wild-type Scc2- or Scc2C-expressing cells were prepared following G1 synchronization, endogenous Scc2 depletion, and release to nocodazole-induced mitotic arrest. Protein A-tagged Sth1 was precipitated, and coprecipitation of Scc2 and Scc4 was analyzed by immunoblotting. (C) Purified Scc2C fails to interact with RSC. The Coomassie-stained gel shows the purified Scc2-Scc4 complex and Scc2C. Equimolar amounts of RSC and either Scc2-Scc4 or Scc2C were mixed. RSC was precipitated by its tandem affinity purification (TAP)-tagged Rsc2 subunit, and copurification of the cohesin loader was analyzed by immunoblotting. (D) Scc4-Scc2N fails to stably interact with RSC. Cell extracts of the indicated strains were obtained as in (B). Protein A-tagged Sth1 was precipitated, and coprecipitation of Scc4 was analyzed by immunoblotting. *, asterisk indicates a non-specific band. See also Figure S5 for characterization of cohesin loader module expression, their chromatin binding, and their ability to promote cohesin loading and sister chromatid cohesion.

Structural and biochemical studies have revealed a functional modularity of the cohesin loader (Chao et al., 2015, Hinshaw et al., 2015, Takahashi et al., 2008). To analyze which cohesin loader module mediates the RSC interaction, we used strains in which endogenous Scc2 could be depleted by combined promoter repression and auxin-mediated degradation. In this background, we expressed either full-length Scc2 or its two separate functional units, Scc2N or Scc2C (Figures 5A and S5A). As expected, full-length Scc2 or Scc2N co-immunoprecipitated Scc4 with equal efficiency whereas Scc2C did not. Scc4 levels in cells lacking Scc2 or expressing Scc2C were noticeably reduced, suggesting that Scc4 must bind to Scc2N for stability (Figure S5B). Although Scc2C is sufficient to catalyze cohesin loading onto naked DNA *in vitro* (Minamino et al., 2018, Murayama and Uhlmann, 2014), it fails to bind chromatin, load cohesin, or support sister chromatid cohesion *in vivo* (Figures S5C and S5D; Chao et al., 2015).

We wondered whether Scc2C is unable to load cohesin onto DNA *in vivo* because it fails to interact with RSC. To address this, we used protein A pull-down of Sth1 in cells expressing either full-length Scc2 or Scc2C. Both Scc2 and Scc4 co-precipitated with Sth1 in cells expressing full-length Scc2, but the interaction was markedly reduced in cells expressing Scc2C (Figure 5B). When using purified proteins, RSC interacted with the Scc2-Scc4 complex but not with Scc2C (Figure 5C). These results suggest that Scc2C is insufficient and that the Scc4-Scc2N module is required to mediate stable RSC interaction.

We next addressed whether the Scc4-Scc2N module is sufficient to bind RSC. We again used cells depleted of endogenous Scc2 but this time expressed Scc2N alongside Scc2 and Scc2C. Sth1 efficiently interacted with Scc4 only in the presence of full-length Scc2. The interaction was greatly reduced in the case of both Scc2N and Scc2C (Figure 5D). This suggests that the Scc4-Scc2N and the Scc2C module make joint contributions to the cohesin loader-RSC interaction.

Because neither Scc4-Scc2N nor Scc2C are by themselves sufficient for cohesin loading *in vivo*, we finally expressed both Scc2N and Scc2C in the same cells. However, even this failed to restore cell growth following depletion of endogenous Scc2 (Figure S5E), suggesting that the Scc4-Scc2N globular head and the Scc2C HEAT repeat module must be linked for cohesin loader function.

### Connecting Scc2C with RSC Replaces Scc4

**Figure 6:**
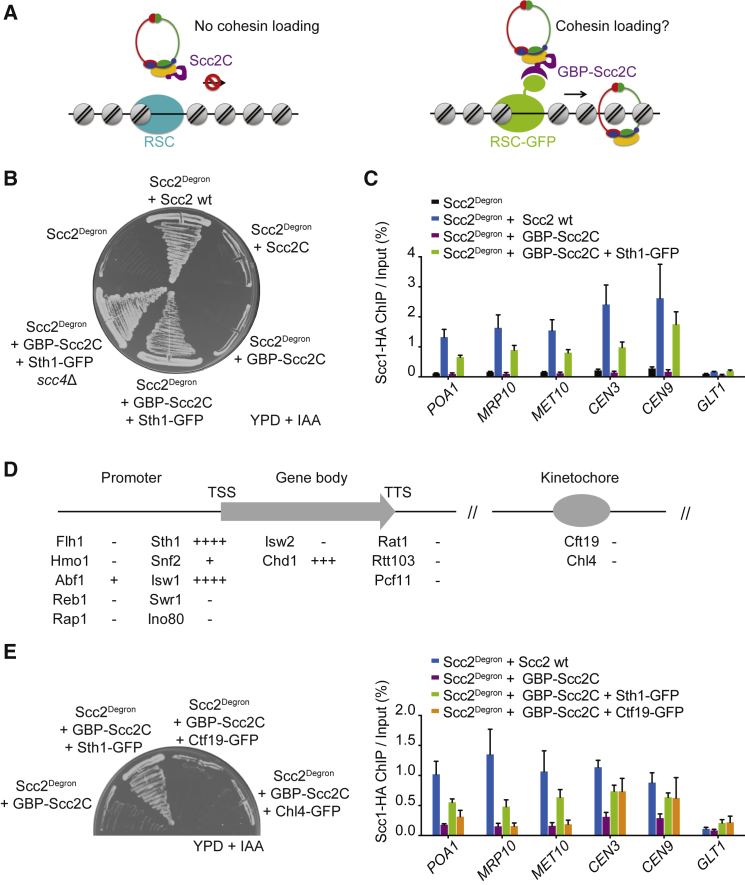
Engineered Cohesin Loading by Chromatin Remodelers and Scc2C (A) Schematic of engineered GBP-Scc2C tethering to candidate chromatin receptors. (B) GBP-Scc2C tethering to Sth1-GFP bypasses the need for Scc2 and Scc4. Scc2^Degron^ cells and Scc2^Degron^ cells expressing the indicated components were streaked on rich YPD medium containing methionine to repress Scc2^Degron^ expression and IAA to promote its degradation. (C) GBP-Scc2C tethering to Sth1-GFP reconstitutes cohesin loading. Chromosomal cohesin levels were assessed in cells of the indicated genotypes following G1 synchronization, endogenous Scc2 depletion, and release into a nocodazole-imposed mitotic arrest. Cohesin was detected by ChIP against Scc1, followed by real-time qPCR at three chromosome arm and two centromere cohesin binding sites and a negative control site. Means and SEM of three independent experiments are shown. Scc2^Degron^ + Scc2 wt and Scc2^Degron^ + GBP-Scc2C + Sth1-GFP, p < 0.01; Scc2^Degron^ + GBP-Scc2C, p not significant; two-way ANOVA test compared with the Scc2^Degron^ strain. (D) Summary of cell growth following endogenous Scc2^Degron^ depletion on YPD + IAA medium and tethering of GBP-Scc2C to the indicated chromatin receptors. Growth was ranked from − to ++++ based on Figure S6C. (E) Tethering GBP-Scc2C to the inner kinetochore restores centromeric cohesin loading but not cell viability. Cell growth and cohesin loading in the indicated strains was analyzed as in (B) and (C). Scc2^Degron^ + Scc2 wt and Scc2^Degron^ + GBP-Scc2C + Sth1-GFP, p < 0.01; Scc2^Degron^ + GBP-Scc2C + Ctf19-GFP, p not significant; two-way ANOVA test compared with the Scc2^Degron^ + GBP-Scc2C strain at chromosome arm sites. Scc2^Degron^ + GBP-Scc2C, p < 0.01; Scc2^Degron^ + GBP-Scc2C + Sth1-GFP and Scc2^Degron^ + GBP-Scc2C + Ctf19-GFP, p not significant; two-way ANOVA test compared with the Scc2^Degron^ + Scc2 wt strain at the two centromeric sites. See also Figure S6 for controls for GBP-Scc2C expression, cohesin loading in the absence of Scc4, and cell growth data following GBP-Scc2C tethering to the various chromatin receptors.

If the main role of Scc4-Scc2N is cohesin loader recruitment to chromatin receptors, then we might be able to replace Scc4-Scc2N with an alternative link between RSC and Scc2C. To test this, we expressed an N-terminal fusion of Scc2C with GFP binding protein (GBP), a single-chain nanobody with high affinity for GFP (Rothbauer et al., 2006; Figure S6A). GBP-Scc2C expression by itself did not rescue cell viability. Strikingly, C-terminal fusion of Sth1 to GFP provided a suitable receptor for GBP-Scc2C to sustain cell proliferation upon Scc2 depletion (Figures 6A and 6B). As could be expected, Scc4 was no longer required for viability of these cells. Chromosomal cohesin levels are depleted in cells expressing only GBP-Scc2C but were restored by Sth1-GFP to at least half of wild-type levels, both along chromosome arms as well as at centromeres (Figure 6C). Cohesin loading was now also independent of Scc4 (Figure S6B). This suggests that the essential role of the Scc4-Scc2N module can be replaced by directing Scc2C to the RSC complex in an alternative way.

### Chromatin Remodelers as Scc2C Receptors

Our ability to reconstitute cohesin loading using GBP-Scc2C allowed us to investigate the requirements at chromosomal cohesin loading sites. In other words, can chromosomal proteins other than RSC serve as functional GBP-Scc2C receptors? To explore this, we fused GFP to the catalytic subunits of the other budding yeast chromatin remodelers (Snf2, Isw1, Isw2, Chd1, Swr1, and Ino80), pioneer transcription factors (Abf1, Reb1, and Rap1), and other regulators that overlap with cohesin loading sites (Fhl1 and Hmo1) (Lopez-Serra et al., 2014). In addition, we included chromatin factors that are expressed at levels comparable with Sth1 but are found at transcription termination sites instead of promoters (Rat1, Rtt103, and Pcf11) (Baejen et al., 2017, Ghaemmaghami et al., 2003, Kulak et al., 2014). Among these alternate GBP-Scc2C receptors, the remodeler Isw1-GFP supported robust cell growth and, to a lesser degree, Chd1-GFP (Figures 6D and S6C). Isw1 and Chd1 are thought to regulate nucleosome spacing during chromatin assembly. Although they are not known to evict nucleosomes, both share with RSC the general mode of mobilizing nucleosomes by DNA translocation (Clapier et al., 2017).

Two additional GFP fusions allowed minimal cell proliferation in conjunction with GBP-Scc2C, those to Snf2 and to Abf1. Snf2 is the catalytic subunit of the SWI/SNF chromatin remodeler, closely related to RSC. However, Snf2 is an order of magnitude less abundant than Sth1 (Ghaemmaghami et al., 2003, Kulak et al., 2014), which could be a reason for the inefficient rescue. Abf1 is a pioneer transcription factor whose binding sites fall into nucleosome-depleted regions that are maintained by RSC (Kubik et al., 2018). Together, this suggests that, among the places tested, Scc2C becomes functional in proximity to a chromatin remodeler. This is consistent with the idea that nucleosome-free DNA is the required substrate for cohesin loading.

Finally, we targeted GBP-Scc2C to the centromere using Ctf19-GFP as a receptor. This restored cohesin loading at the centromere to around half the levels seen in wild-type cells. Cohesin levels remained low along chromosomes arms (Figure 6E). Despite substantial cohesin loading at centromeres, GBP-Scc2C recruitment to Ctf19-GFP or to Chl4-GFP, another component of the Ctf19 inner kinetochore complex, did not support cell growth. This opens the possibility that RSC-mediated cohesin loading along chromosome arms, as well as at centromeres, is required for survival.

## Discussion

The chromosomal loading sites of the cohesin complex at centromeres and at promoters of actively transcribed genes have been known for some time (Kagey et al., 2010, Lengronne et al., 2004, Zuin et al., 2014), but the chromatin characteristics underlying these locations were incompletely understood. Previous work pointing to a role for the yeast RSC chromatin remodeler (Lopez-Serra et al., 2014) left unanswered how RSC promotes cohesin loading. Here we describe a dual but closely linked role of RSC during cohesin loading. First, RSC acts as the chromatin receptor for the Scc2-Scc4 complex, and second, chromatin remodeling makes nucleosome-free DNA accessible for the cohesin loading reaction. These findings have implications for how cohesin accesses DNA in the context of chromatin.

### RSC as a Chromatin Receptor for the Cohesin Loader

Our characterization of an ATPase-deficient RSC complex revealed its role as an Scc2-Scc4 complex receptor on chromatin that is independent of RSC’s role in chromatin remodeling. Direct protein interactions exist between RSC and the cohesin loader, involving both the Scc2 and Scc4 subunits, as well as direct interactions with the cohesin complex. The RSC complex consists of 19 subunits (Clapier et al., 2017), providing opportunities for multiple protein contacts. The details of these interactions and their implications for cohesin loading will be fertile ground for further investigation.

An initial cohesin loader recruitment role for the RSC complex, independent of chromatin remodeling, is consistent with observations that forced nucleosome positioning into a previously open *RPL19B* gene promoter does not impede Scc2-Scc4 recruitment (Lopez-Serra et al., 2014). The determinants of cohesin loading sites thus appear to be those of the RSC complex, which, in turn, uses a still incompletely understood combinatorial mechanism to find its target sites. This includes specificities for AT-rich sequences, acetylated histone H3 tails, and help from pioneer transcription factors, all of which are found at cohesin loading sites (Kubik et al., 2018). Peak calling in RSC and Scc2-Scc4 chromatin immunoprecipitation experiments requires thresholding that will have limited our knowledge to the most robust binding sites (Kagey et al., 2010, Kubik et al., 2018, Lopez-Serra et al., 2014, Zuin et al., 2014). Cohesin loading might take place at additional promoters and other places in the genome where Scc2-Scc4 and RSC occupancy falls below detection thresholds.

The important role of the Scc4-Scc2N module for cohesin loading onto chromosomes *in vivo*, but not onto naked DNA *in vitro*, could be explained by its role as a RSC adaptor. Although Scc4 is essential for cohesin loading in most instances (Bernard et al., 2006, Ciosk et al., 2000, Watrin et al., 2006), a recent report suggests that human haploid HAP-1 cells are able to proliferate without Scc4 (Haarhuis et al., 2017). Interactions that both Scc2 and cohesin make with the RSC complex, or additional chromatin receptors in human cells, could make up for this loss. Support for this interpretation comes from a recent genetic study in budding yeast. An Scc2 mutation that increases its affinity for cohesin also makes Scc4 dispensable (Petela et al., 2018). Given the three-way interactions between cohesin, its loader, and the RSC complex, increased affinity between one pair could compensate for loss of another, thus allowing cohesin loading without Scc4. At budding yeast centromeres, cohesin acts as a cofactor for Scc2-Scc4 recruitment (Fernius et al., 2013), providing further evidence for the interdependence of cohesin and cohesin loader recruitment. Although cohesin loader recruitment to centromeres is augmented by the Ctf19 inner kinetochore complex, cohesin loading remains dependent on RSC, which is enriched at centromeres (Hsu et al., 2003).

### Nucleosome-free DNA as the Substrate for SMC Complex Loading

In addition to being a receptor for the cohesin loader, the RSC ATPase is required to facilitate cohesin loading. This suggests that nucleosome sliding and/or eviction is part of the cohesin loading reaction. In a reconstituted *in vitro* reaction, Scc2-Scc4 stimulates cohesin loading onto naked DNA but not nucleosomal DNA. Thus, nucleosome-free DNA, provided by the RSC complex, is likely also a required substrate for cohesin loading *in vivo*. Among the chromatin remodelers, the RSC complex within the SWI/SNF family is specialized in facilitating chromatin access (Clapier et al., 2017). However, there is no exclusive relationship between RSC and cohesin loading. Scc2C fusion to other SWI/SNF, ISWI, or CHD remodelers, representing three of the four eukaryotic chromatin remodeler families, provide alternative means to load cohesin. We note that RSC depletion greatly reduces, but does not abolish, cohesin loading, consistent with the possibility of promiscuous help from other remodelers. Only Scc2C fusion to INO80 family members was unable to support cell viability. This could have been for trivial technical reasons or it could be that the specific function of INO80 remodelers in nucleosome editing limits their ability to support cohesin loading.

Do other SMC complexes require similar nucleosome-free regions for their chromatin association? Condensin loading sites in budding yeast coincide with those of cohesin, even though no direct protein interaction is known between condensin and the cohesin loader (D’Ambrosio et al., 2008). Rather, condensin interacts with TBP and TFIIIC transcription factor complexes (Haeusler et al., 2008, Kim et al., 2016). Although the chromatin receptors differ, a common requirement for accessible nucleosome-free DNA in open chromatin might unite the SMC complexes. Condensin also loads at open promoter regions in fission yeast, *C. elegans*, and human cells, whereas the fission yeast RSC complex has been implicated in the loading of both cohesin and condensin onto chromosomes (Kotomura et al., 2018, Kranz et al., 2013, Sutani et al., 2015, Toselli-Mollereau et al., 2016). Given that DNA has to pass through protein-protein interfaces to enter an SMC ring (Murayama and Uhlmann, 2015), it is plausible that nucleosomes pose a steric hindrance.

### Implications for Cohesin Loading in Higher Eukaryotes

Tethering the Scc2C cohesin loader module to various chromatin receptors allowed us to probe possible locations for cohesin loading. RSC fusion sustained cell growth and supported cohesin loading along both chromosome arms as well as at centromeres. These findings do not exclude the existence of additional or alternative chromatin receptors. Specialized receptors might serve other cohesin-dependent processes, such as DNA repair. The human NIPBL^Scc2^ cohesin loader subunit engages with the heterochromatin protein HP1γ to promote cohesin loading at sites of double-stranded DNA breaks (Bot et al., 2017). On transcriptionally inactive chromatin in *Xenopus* oocyte extracts, the pre-replicative complex serves as a cohesin entry point (Takahashi et al., 2008), which, in turn, might be linked to nucleosome-free regions. An additional reported interaction of the cohesin loader with the MCM helicase facilitates cohesin loading during S phase in HeLa cells (Zheng et al., 2018).

Circumstantial evidence links RSC orthologs to cohesin loading in higher eukaryotes. Depletion of BAF180, a subunit of the mammalian PBAF complex, leads to sister chromatid cohesion defects both in mouse embryonic stem cells as well as in human cell lines (Brownlee et al., 2014). Furthermore, mutations in NIPBL are the cause of Cornelia de Lange syndrome, a congenital disorder whose clinical features are thought to be the collective outcome of gene expression changes during development (Krantz et al., 2004). The closely related clinical features of Coffin-Siris syndrome result from mutations in subunits of the human BAF chromatin remodeler (Parenti et al., 2017), suggesting functional overlap between the human cohesin loader and BAF. We note that a human ISWI chromatin remodeling complex has also been implicated in cohesin loading onto chromosomes, as has been the human mediator complex that is found at active promoters (Hakimi et al., 2002, Kagey et al., 2010). Whether a subset or all of these chromatin remodelers and transcription factors are receptors for the human cohesin loader remains to be explored. The role of chromatin remodeling during loading of the human cohesin complex onto chromosomes and its link to human disease is an important area for further investigations.

## STAR★Methods

### Key Resources Table

**Table:** 

REAGENT or RESOURCE	SOURCE	IDENTIFIER
**Antibodies**		

Rabbit polyclonal anti-Sth1	Gift from Cairns Lab	N/A
Mouse monoclonal anti-Scc1 (362 D11B10) (budding yeast)	Gift from Shirahige Lab	N/A
Mouse monoclonal anti- α-tubulin (TAT-1)	Cell Services Science Technology Platform, The Francis Crick Institute	N/A
Mouse monoclonal anti-V5	Bio-Rad	Cat# MCA1360
Mouse monoclonal anti-HA (F7)	Santa Cruz	Cat# sc-7392
Mouse monoclonal anti-HA (12CA5)	Cell Services, The Francis Crick Institute	N/A
Rabbit Peroxidase Anti-Peroxidase	Sigma-Aldrich	Cat# P1291
Anti-mouse IgG (HRP-conjugated)	GE Healthcare	Cat# NA931
Anti-rabbit IgG (HRP-conjugated)	GE Healthcare	Cat# NA934
Anti-AID Tag (IAA17) Protein	2B Scientific	Cat# CAC-APC004AM

**Chemicals, Peptides, and Recombinant Proteins**		

α-factor	Peptide Chemistry Science Technology Platform, The Francis Crick institute	N/A
Nocodazole	Sigma-Aldrich	Cat# M1404
Indole-3-acetic acid (IAA)	Sigma-Aldrich	Cat# I3750
G418	Sigma-Aldrich	Cat# G8618
Hygromycin B	Invitrogen	Cat# 10687010
Formaldehyde solution	Sigma-Aldrich	Cat# 252549
Phenylmethylsulfonyl fluoride (PMSF)	Sigma-Aldrich	Cat# P7626
Pefabloc SC	Roche	Cat# 11 429 876 001
cOmplete EDTA-Free Protease Inhibitor Cocktail	Sigma-Aldrich	Cat# 04693132001
Zymoliase 100T	MP	Cat# 320931
Micrococcal Nuclease	ThermoFisher	Cat# EN0181
Proteinase K	ThermoFisher	Cat# EO0491
Benzonase Nuclease	Sigma-Aldrich	Cat# E1014
RNase A	Sigma-Aldrich	Cat# 10109169001
Protein Assay Dye	Bio-Rad	Cat# 5000006
Propidium iodide solution	Sigma-Aldrich	Cat# P4864
InstantBlue Coomassie Protein Stain	Expedeon	Cat# HG773010
GelRed Nucleic Acid Gel Stain	Biotium	Cat# 41003-1
PowerUp SYBR Green Master Mix	ThermoFisher	Cat# A25742
AcTEV Protease	ThermoFisher	Cat# 12575015
PreScission Protease	GE Healthcare	Cat# 27084301
ATP	Sigma-Aldrich	Cat# A2383
Phosphocreatine di(tris) salt	Sigma-Aldrich	Cat# P1937
Creatine Kinase	Sigma-Aldrich	Cat# 10127566001
PstI	New England Biolabs	Cat# R0140S
Isopropyl β-D-1-thiogalactopyranoside (IPTG)	Sigma-Aldrich	Cat# 10724815001
Flag peptide	Peptide Chemistry Science Technology Platform, The Francis Crick institute	N/A
Budding yeast cohesin (Smc1-Smc3-Scc1-Scc3)	Minamino et al., 2018	N/A
Budding yeast Scc2-Scc4	Minamino et al., 2018	N/A
Budding yeast Scc2C	Minamino et al., 2018	N/A
Budding yeast Histones	Kurat et al., 2017	N/A
Budding yeast Nap1	Kurat et al., 2017	N/A
Budding yeast ISW1A	Kurat et al., 2017	N/A
Budding yeast RSC	Wittmeyer et al., 2004	N/A

**Critical Commercial Assays**		

InFusion HD cloning kit	Clontech Laboratories	Cat# 639634
CloneAmp HiFi PCR Premix	Clontech Laboratories	Cat# 639298
Q5 Site-Directed Mutagenesis Kit	New England Biolabs	Cat# E05545
Dynabeads Protein A	ThermoFisher	Cat# 10002D
Dynabeads M-270 Epoxy	ThermoFisher	Cat# 14302D
Rabbit immunoglobulin G (IgG)	Sigma-Aldrich	Cat# I5006
ECL Prime Western Blotting Detection Regent	GE Healthcare	Cat# RPN2232
Human IgG-Agarose	Sigma-Aldrich	Cat# A6284-5ML
Calmodulin Affinity Resin	Agilent Technologies	Cat# 214303
HiTrap Heparin HP 1 ml	GE Healthcare	Cat# 17040601
Superdex 200 Increase 10/300 GL	GE Healthcare	Cat# 28990944
Superose 6, 10/300 GL	GE Healthcare	Cat# 17517201
HiTrap Heparin HP 5 ml	GE Healthcare	Cat# 17040701
HiLoad 16/600 Superdex 200	GE Healthcare	Cat# 28-9893-35
Glutathione Sepharose 4B	GE Healthcare	Cat# 17075601
Mono Q 5/50 GL, 1 ml	GE Healthcare	Cat# 17516601
ANTI-FLAG M2 Affinity Gel	Sigma-Aldrich	Cat# A2220
Slide-A-Lyzer Dialysis Cassettes, 3.5K MWCO, 12 mL	ThermoFisher	Cat# 66110
MicroSpin S-400 HR columns	GE Healthcare	Cat# 27514001
Amicon Ultra-4 centrifuge filter unit, 10 NMWL	MERCK MILLIPORE	Cat# UFC801024
Vivaspin 20 centrifugal concentrator, 100,000 MWCO	Sartorius	Cat# VS2042

**Experimental Models: Organisms/Strains**		

All *Saccharomyces cerevisiae* strains used in this study are listed in Table S1	Lab stock and this study	N/A
*Escherichia coli* DH5α competent cells	New England Biolabs	Cat# C2987U

**Oligonucleotides**		

All oligonucleotides used for qPCR are listed in Table S2.	N/A	N/A

**Recombinant DNA**		

All plasmid DNA used in this study are listed in Table S3.	N/A	N/A

**Software and Algorithms**		

Snapgene v2.6	GSL Biotech	N/A
FlowJo v10.1	FlowJo	N/A
ImageQuant TL v8.1	GE Healthcare	N/A
ImageJ v1.50c	NIH, USA	N/A

**Deposited Data**		

MNase sequencing data	This study	GEO: GSE117881
Unprocessed gel images presented in this manuscript	This study	https://doi.org/10.17632/34fry4t69p.1

### Contact for Reagent and Resource Sharing

Further information and requests for resources and reagents should be directed to and will be fulfilled by the Lead Contact, Frank Uhlmann (frank.uhlmann@crick.ac.uk).

### Experimental Model and Subject Details

All *Saccharomyces cerevisiae* yeast strains used in this study were of the W303 background and are listed in Table S1. Cells were cultured at 25°C. α-factor was used at a concentration of 7.5 μg/ml, nocodazole at 6 μg/ml and indole-3-acetic acid (IAA) acid at 88 μg/ml.

### Method Details

#### Yeast Strains and Culture

Epitope tagging of endogenous genes and gene deletions were performed by gene targeting using polymerase chain reaction (PCR) products. Cells were grown in rich YP medium or in complete synthetic medium (CSM) lacking methionine, supplemented with 2% glucose. To deplete Sth1 or Scc2, their gene promoters were replaced with the methionine-repressible *MET3* promoter and their C terminus fused to an auxin-inducible degron (Nishimura et al., 2009). Cells were grown in medium lacking methionine, arrested 1.5 hour with α-factor, and shifted to YP medium containing methionine and indole-3-acetic acid (IAA) in addition to α-factor for 2 hours, before release from α-factor block into synchronous cell cycle progression until reaching a nocodazole-imposed mitotic arrest. Samples for analysis were taken 120 minutes after α-factor release. Expression of wild-type Sth1 or Sth1^K501R^ was accomplished by cloning the *STH1* gene under control of its own promoter into the yeast-*E. coli* shuttle vector YIplac204 including a Pk, HA or Protein A epitope tag. The Sth1^K501R^ mutation was introduced by site directed mutagenesis using the Q5 Site-Directed Mutagenesis Kit (New England Biolabs). The resulting plasmids were integrated into the budding yeast genome at the *TRP1* locus. Expression of wild-type Scc2, Scc2C or Scc2N was achieved by cloning the *SCC2* gene under control of its own promoter into the yeast-*E. coli* shuttle vector pRS303, including a Pk epitope tag for detection at the C terminus. Scc2C or Scc2N fragments were derived by deleting part of the Scc2 sequence by site directed mutagenesis. The resulting plasmids were integrated into the budding yeast genome at the *HIS3* locus.

#### Yeast Molecular Biology Techniques

##### Immunoblotting

Protein extracts for immunoblotting were prepared following cell fixation using trichloroacetic acid and separated by SDS-polyacrylamide gel electrophoresis before transfer to nitrocellulose membranes. Antibodies used for detection are listed in the Key Resources Table and were visualized using ECL reagents and film (GE Healthcare).

##### FACS analysis of DNA content

Cells were fixed in cold 70% ethanol overnight, then treated with 0.1 mg/ml RNase A in RNase buffer (50 mM Tris-HCl pH 7.5) at 37°C for 2 hours. DNA was stained with 50 μg/ml propidium iodide in FACS buffer (200 mM Tris-HCl pH 7.5, 211 mM NaCl, 78 mM MgCl_2_). Samples were sonicated and diluted in 50 mM Tris-HCl pH 7.5. 10,000 cells per sample were analyzed using a FACSCalibur cell analyzer (BD Biosciences) and the data files were curated using FlowJo.

##### Protein interaction analysis

Cell extracts were prepared in EBX buffer (50 mM HEPES-KOH pH 7.5, 100 mM KCl, 2.5 mM MgCl_2_, 10% glycerol, 0.25% Triton X-100, 1 mM DTT, protease inhibitors and benzonase) using glass beads breakage in a cooled Multi-Beads Shocker (Yasui Kikai). Extracts were cleared by centrifugation, precleared and incubated with either IgG coated Dynabeads (ThermoFisher) for Protein A pulldown or with Protein A Dynabeads previously ligated to the respective epitope-specific antibody. Beads were extensively washed and elution was carried out in SDS-PAGE loading buffer.

##### Quantitative ChIP

Chromatin immunoprecipitation was performed as previously described (Katou et al., 2006). Briefly, cells were fixed with formaldehyde and harvested. Protein extracts were prepared and disrupted by sonication. DNA fragments cross-linked to the tagged protein of interest were enriched by immunoprecipitation. After reversal of the cross-links, DNA both from immunoprecipitates and from whole cell extract was purified and quantified using the PowerUP SYBR Green Master Mix (ThermoFisher) and a Quant Studio 12K Real-Time PCR System (Thermo Fisher). All primer sequences used are listed in Table S2.

##### Sister chromatid cohesion assay

Cells carrying a GFP-marked *URA3* locus were synchronized in G1 using α-factor and released into a nocodazole-imposed mitotic arrest. Cells were fixed with ice-cold 100% ethanol and imaged using a DeltaVision wide-field fluorescence microscope (GE Healthcare). z stacks with 15 images at 0.1 μm intervals were acquired and merged by maximum intensity projection. Quantification of the percentage of cells showing two separated GFP foci was performed using ImageJ.

#### Nucleosome Positioning Analysis

Mononucleosomal DNA isolation was performed as described (Lantermann et al., 2009). Cells were fixed with formaldehyde, cell walls digested with Zymolase 100T and unprotected DNA was digested with 30 U MNase for 20 minutes at 37°C. DNA was purified, size separated by agarose gel electrophoresis and the band corresponding to mononucleosomal DNA was excised and processed for sequencing. 100 bp paired end sequencing of MNase-resistant DNA was performed on either the Illumina HiSeq 2500 or 4000 platforms to generate ∼100 million reads. Raw reads from each sample were adaptor-trimmed using cutadapt (version 1.9.1) (Martin, 2011) with parameters -a: AGATCGGAAGAGC, -A: AGATCGGAAGAGC, minimum-length = 25, quality-cutoff = 20. BWA (version 0.5.9-r16) (Li and Durbin, 2009) with default parameters was used to perform genome-wide mapping of the adaptor-trimmed reads to the yeast sacCer3 genome. Alignments were filtered to remove read pairs that were discordant, mapped to different chromosomes, ambiguously mapped, had an insert size outside the range 120-200 bp, or more than 4 mismatches in any read. Sample-level smoothed coverage tracks for nucleosome profile plots were generated with the DANPOS2 dpos command (version 2.2.2) (Chen et al., 2015) with parameters paired: 1, span: 1, smooth width: 20, width: 40, count: 10,000,000. The MNase, histone H4-ChIP data of *in vivo* formaldehyde-crosslinked cells (Zhang et al., 2011) were used as the reference dataset for +1 nucleosome dyad locations.

#### Protein Purification

Histones, Nap1 and ISW1A were expressed and purified as described in (Kurat et al., 2017). Histones were expressed in bacteria after Isopropyl β-D-1-thiogalactopyranoside (IPTG) induction, the cells were broken by sonication and the extract clarified by ultracentrifugation. Histone octamers were then purified by sequential column chromatography, 5 mL HiTrap Heparin and Superdex 200 Increase 16/600 GL (GE Healthcare). GST-tagged Nap1 was expressed in bacteria by IPTG induction, cells were broken by sonication and the extract clarified by ultracentrifugation. The protein was bound to glutathione agarose beads, Nap1 was released from beads by digestion with PreScission Protease (GE Healthcare), dialyzed and purified on a 1 mL HiTrap Q column (GE Healthcare). ISW1A was purified from yeast cells expressing an endogenously Flag epitope-tagged Ioc3 subunit. Cells were grown in YPD to stationary phase and disrupted in a cryogenic grinder under liquid nitrogen. Ioc3-Flag was bound to anti-Flag M2 affinity gel (Sigma-Aldrich), eluted with Flag peptide and further purified using a HiTrap Q column.

Cohesin and the cohesin loader were purified following overexpression under control of galactose-inducible promoters in budding yeast as described in detail elsewhere (Minamino et al., 2018). Briefly, cells were grown in YP medium containing 2% raffinose and protein expression was induced by addition of 2% galactose. Cells were disrupted in a cryogenic grinder under liquid nitrogen, the frozen cell powder was thawed and the lysate was clarified by ultracentrifugation. The lysis buffer contained 5 U/ml Benzonase (Sigma) and 1 μg/ml RNase A (Sigma). The complexes were purified by sequential column chromatography, protein A affinity adsorption on IgG-agarose (Sigma) followed by PreScission Protease elution, HiTrap Heparin HP and Superose 6 Increase 10/300 GL (cohesin) or Superdex 200 Increase 10/300 GL (cohesin loader; GE Healthcare).

RSC was purified from budding yeast cells expressing an endogenously TAP-tagged Rsc2 subunit, as described (Wittmeyer et al., 2004). Cells were grown in YPD to stationary phase and disrupted in a cryogenic grinder under liquid nitrogen. The frozen cell powder was thawed and the lysate was clarified by ultracentrifugation. The lysis buffer was supplemented with 5 U/ml Benzonase and 1 μg/ml RNase A. The complex was purified by binding to IgG-agarose, followed by TEV protease elution, then bound to calmodulin beads in the presence of calcium, eluted in the presence of EGTA and dialyzed.

#### Co-precipitation of purified proteins

50 nM Scc2-Scc4, 50 nM cohesin and 50 nM RSC were mixed in 50 μL of IP buffer (25 mM Tris-HCl pH 7.5, 0.5 mM TCEP, 100 mM NaCl, 2.5 mM MgCl_2_, 0.2% Triton X-100, 5% glycerol and 125 U/ml Benzonase) and incubated at 25°C for 15 minutes. After placing on ice for 15 minutes, the binding mixtures were transferred to either antibody-coated, protein A-conjugated magnetic beads for cohesin loader and cohesin precipitation or calmodulin beads in the presence of calcium for RSC precipitation, and rocked 2 hours at 4°C. The beads were washed three times with IP buffer and once with IP buffer containing 300 mM NaCl. The bound proteins were eluted either in SDS-polyacrylamide gel electrophoresis (SDS-PAGE) loading buffer, or in the presence of EGTA in the case of RSC.

#### Nucleosome Assembly

Yeast histones (6.86 μg), Nap1 (14.61 μg) and ISW1A (384 ng) were mixed in a 40 μL reaction volume in buffer containing 10 mM HEPES-KOH pH 7.5, 50 mM KCl, 5 mM MgCl_2_, 0.5 mM EDTA, 10% glycerol and 0.1 mg/ml BSA and incubated for 30 minutes on ice. Then, 45 mM creatine phosphate, 3 mM ATP, 6 μg creatine phosphate kinase and 1 μg plasmid DNA were added and incubated at 30°C for 5 hours. Following chromatin assembly, the buffer was exchanged to 10 mM HEPES-KOH pH 7.5, 50 mM KCl and 10% glycerol using an illustra MicroSpin S-400 HR Column (GE Healthcare).

#### *In Vitro* Cohesin Loading

The *in vitro* cohesin loading assay was performed as described in (Minamino et al., 2018), with adaptations for use with a chromatin template. 15 nM cohesin tetramers and 30 nM cohesin loader were mixed with 30 ng of either free or chromatinized plasmid DNA in a 15 μL reaction in buffer containing 35 mM Tris-HCl pH 7.0, 35 mM KCl, 20 mM NaCl, 0.05 mM MgCl_2_, 0.003% Tween, 1 mM tris[2-carboxyethyl]phosphine (TCEP) and 10% glycerol. Reactions were initiated by addition of 2.5 mM ATP and incubated for 1 hour at 29°C. Cohesin-DNA complexes were recovered by cohesin immunoprecipitation, beads were washed with high salt buffer (35 mM Tris pH 7.5, 750 mM NaCl, 10 mM EDTA, 5% Glycerol, 0.35% Triton X-100), DNA was released from beads by proteinase K digestion and was analyzed by agarose-gel electrophoresis. Gels were stained with GelRed and bands were visualized and quantified using an Amersham Imager 600 (GE Healthcare).

### Data and Software Availability

The MNase sequencing data generated in this study has been deposited with the Gene Expression Omnibus https://www.ncbi.nlm.nih.gov/geo/ with the accession number GSE117881. Unprocessed gel images presented in this manuscript can be found at https://doi.org/10.17632/34fry4t69p.1

## Supplemental Information

Document S1. Figures S1–S6 and Tables S2 and S3

Table S1. Yeast Strains Used in This Study, Related to STAR Methods

Document S2. Article plus Supplemental Information
