# Supplementary material for: A Role for Chromatin Remodeling in Cohesin Loading onto Chromosomes
Source: Mol Cell. 2019 May 16;74(4):664–673.e5. doi: 10.1016/j.molcel.2019.02.027 (PMC6527865; doi:10.1016/j.molcel.2019.02.027)
Supplement: Document S1. Figures S1–S6 and Tables S2 and S3 [file mmc1.pdf]

Molecular Cell, Volume *74*

Supplemental Information

A Role for Chromatin Remodeling

in Cohesin Loading onto Chromosomes

Sofía Muñoz, Masashi Minamino, Corella S. Casas-Delucchi, Harshil Patel, and Frank Uhlmann

Figure S1

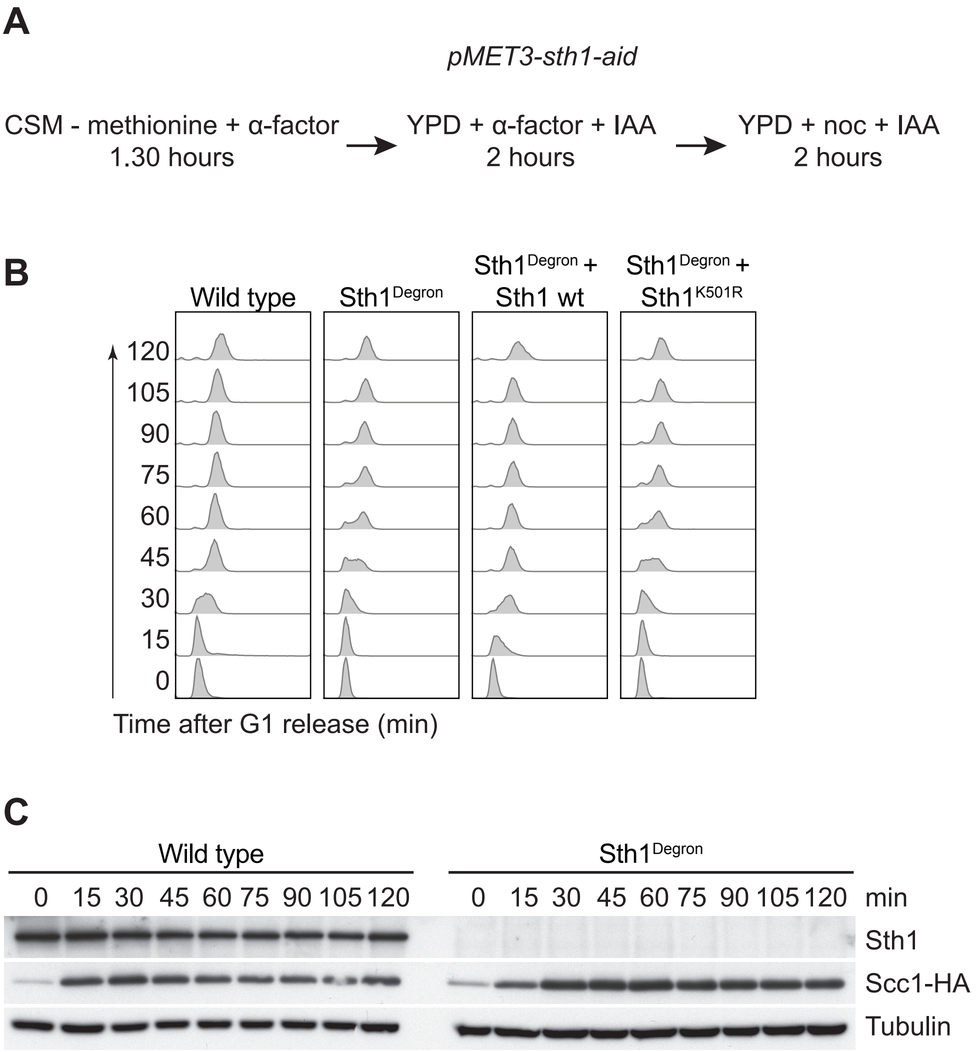

A
pMET3-sth1-aid
CSM - methionine + α-factor
1.30 hours
YPD + α-factor + IAA
2 hours
YPD + noc + IAA
2 hours
B
Wild type
Sth1Degron
Sth1Degron +
Sth1 wt
Sth1Degron +
Sth1K501R
120
105
90
75
60
45
30
15
0
Time after G1 release (min)
C
Wild type
Sth1Degron
0 15 30 45 60 75 90 105 120
0 15 30 45 60 75 90 105 120
min
Sth1
Scc1-HA
Tubulin

Figure S1. Cell Synchronization Protocol Used in This Study, Related to Figure 1

(A) Schematic of the experimental procedure followed in the *in vivo* experiments contained in this study to deplete either endogenous Sth1 or Scc2.

(B) FACS analysis of DNA content during the experiment shown in Figure 1B. A wild type control is included as well as the $Sth1^{Degron}$ strain in which Sth1 was depleted, next to $Sth1^{Degron}$ strains expressing either wild type Sth1 or $Sth1^{K501R}$ from an ectopic locus.

(C) Time course analysis Scc1 levels in strains expressing or depleted of Sth1. Samples for immunoblotting were taken at 15 minute intervals following release from G1 arrest into nocodazole-containing medium.

Figure S2

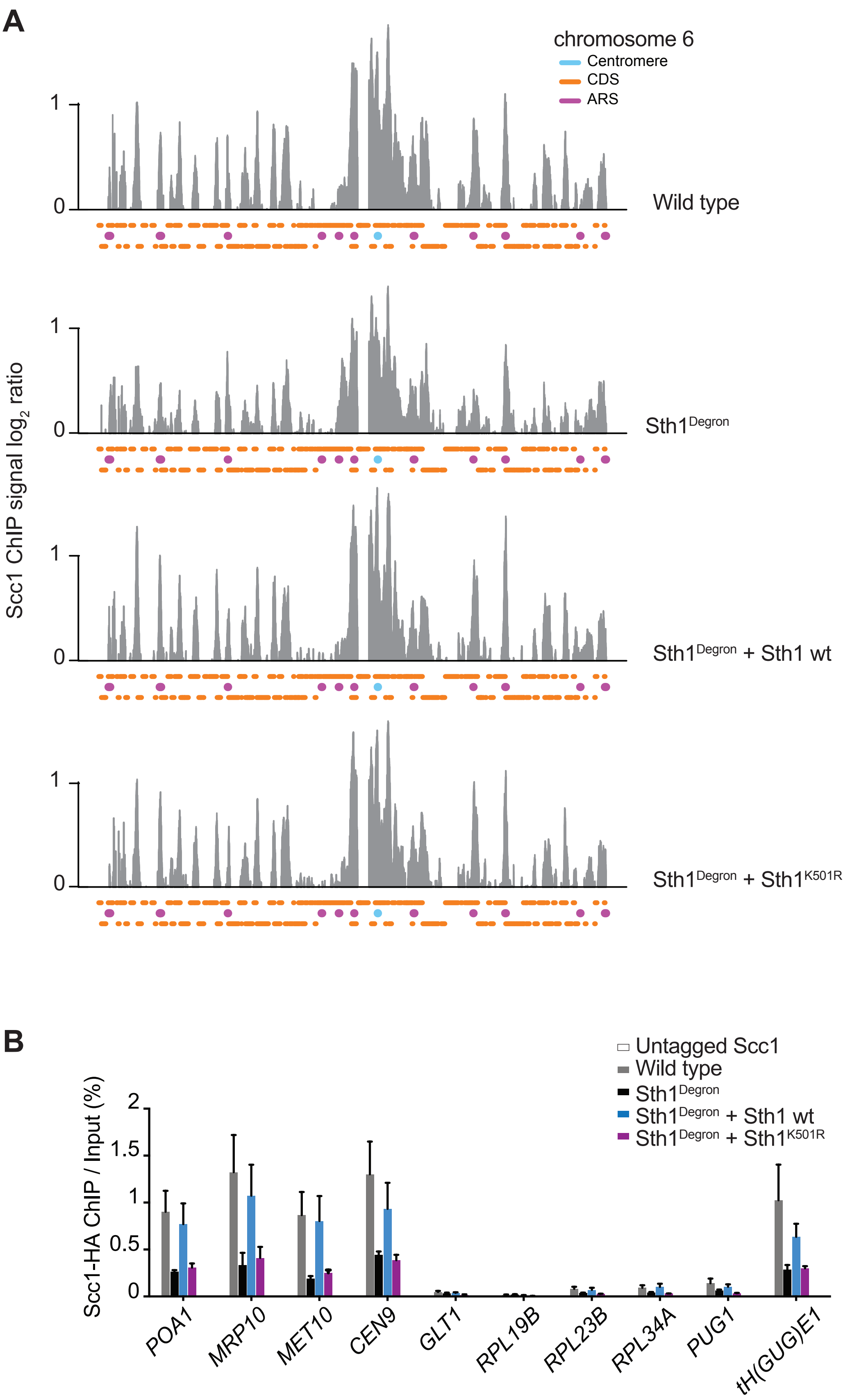
A
chromosome 6
Centromere
CDS
ARS
Scc1 ChIP signal log2 ratio
1
0
Wild type
Sth1Degron
Sth1Degron + Sth1 wt
Sth1Degron + Sth1K501R
B
Untagged Scc1
Wild type
Sth1Degron
Sth1Degron + Sth1 wt
Sth1Degron + Sth1K501R
Scc1-HA ChIP / Input (%)
2
1.5
1
0.5
0
POA1
MRP10
MET10
CEN9
GLT1
RPL19B
RPL23B
RPL34A
PUG1
tH(GUG)E1

Figure S2. Cohesin Distribution along Chromosomes following Sth1 Depletion, Related to Figure 1

(A) Cells of the indicated genotypes were synchronized in G1, endogenous Sth1 was depleted, and released into a nocodazole-imposed mitotic arrest. ChIP analysis against HA epitope-tagged Scc1 was performed and chromatin immunoprecipitates were PCR amplified and hybridized to Affymetrix GeneChip *S. cerevisiae* tiling 1.0 R arrays. Signal intensities, relative to a whole-genome DNA sample, are shown along chromosome 6. Centromeres, replication origins and coding regions are indicated. Note that due to the PCR amplification step, the cohesin pattern does not contain quantitative information about the amount of chromosomal cohesin.

(B) as in (A) but Scc1-HA levels at three chromosome arm cohesin binding sites *(POA1*, *MRP10* and *MET10*), one centromere (*CEN9*), a negative control site (*GLT1*) and five cohesin loading sites (*RPL19B, RPL23B*, *RPL34A*, *PUG1*, *tH(GUG)E1*) were assessed by ChIP followed by quantitative real-time PCR. Means and SEM of three independent experiments are shown to allow a quantitative comparison of chromosomal cohesin levels. DNA recovered by ChIP from an untagged strain was also quantified but remained below the visualization limit of this graph.

Figure S3

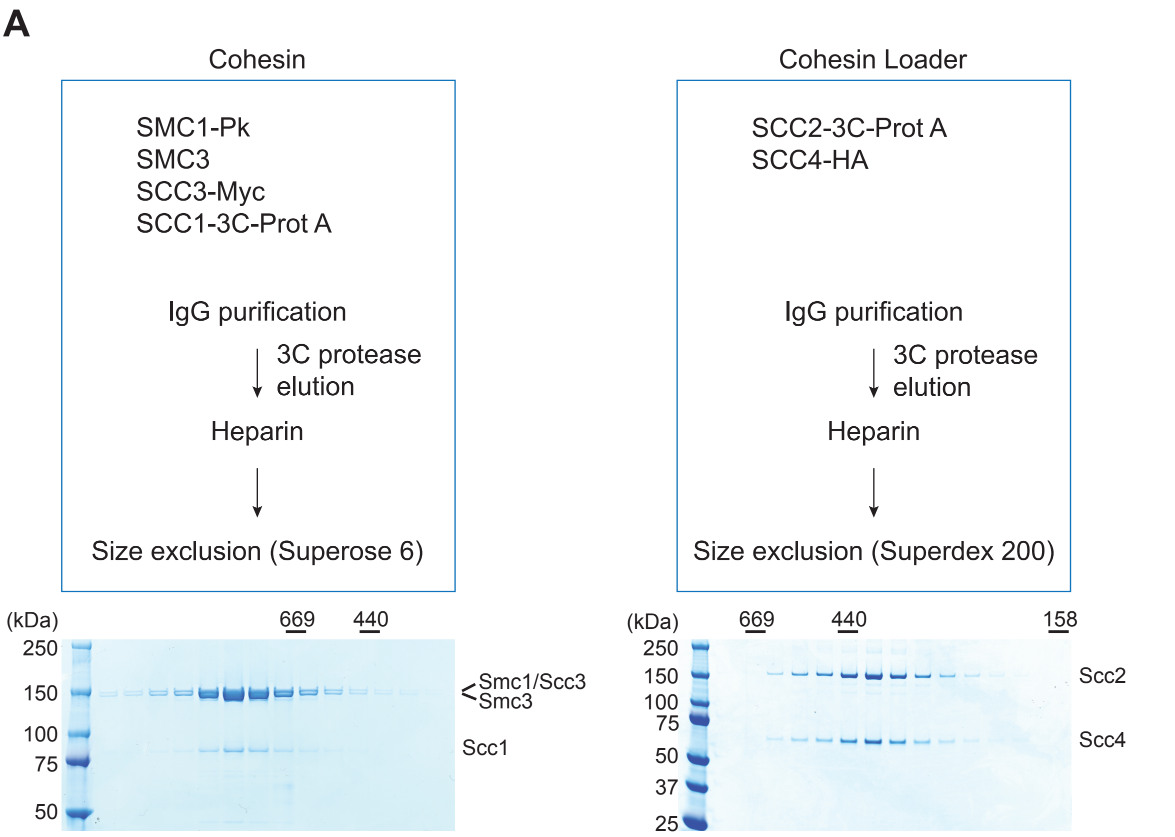
A
Cohesin
SMC1-Pk
SMC3
SCC3-Myc
SCC1-3C-Prot A
IgG purification
3C protease elution
Heparin
Size exclusion (Superose 6)
Cohesin Loader
SCC2-3C-Prot A
SCC4-HA
IgG purification
3C protease elution
Heparin
Size exclusion (Superdex 200)
(kDa)
250
150
100
75
50
669
440
Smc1/Scc3
Smc3
Scc1
(kDa)
250
150
100
75
50
37
25
669
440
158
Scc2
Scc4

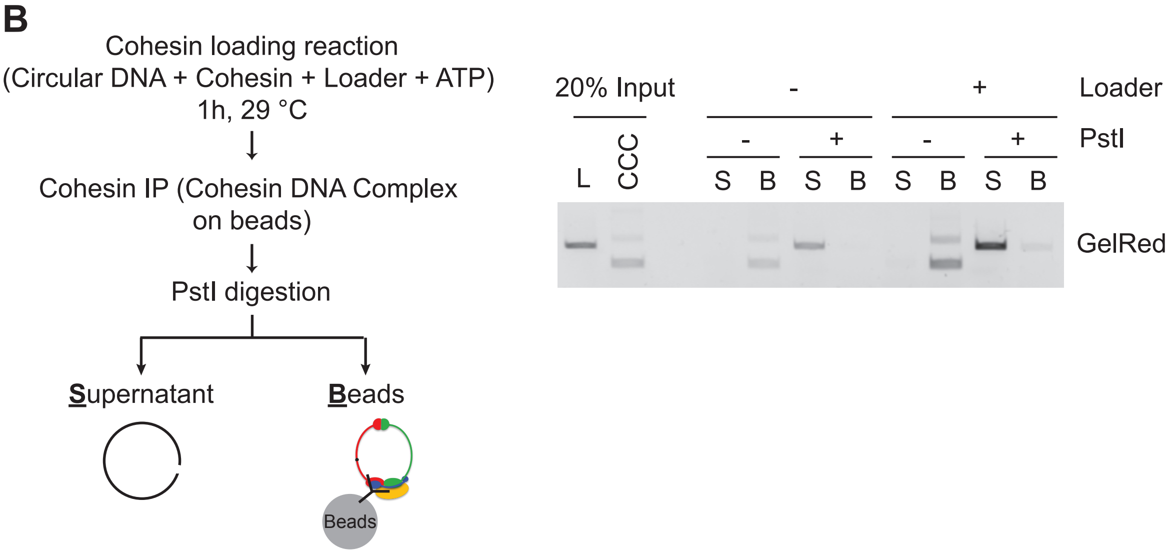
B
Cohesin loading reaction
(Circular DNA + Cohesin + Loader + ATP)
1h, 29 °C
Cohesin IP (Cohesin DNA Complex on beads)
PstI digestion
Supernatant
Beads
Beads
20% Input
L
CCC
Loader
-
+
PstI
-
+
-
+
S
B
S
B
S
B
S
B
GelRed

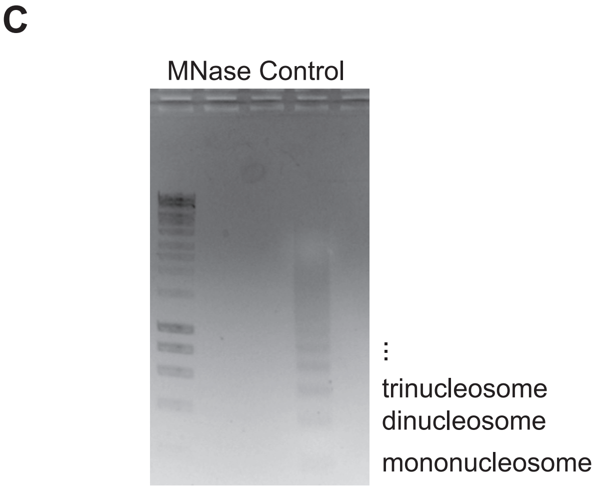
C
MNase Control
trinucleosome
dinucleosome
mononucleosome

Figure S3. Biochemical Reconstitution of Topological DNA Binding by the Budding Yeast Cohesin Ring, Related to Figure 3

(A) Schematic of the cohesin and cohesin loader complex purification procedures. The peak fractions from the final gel filtration steps were analyzed by SDS-PAGE followed by Coomassie Blue staining.

(B) Schematic and gel image of a cohesin loading experiment, followed by DNA release by DNA linearization using the PstI restriction endonuclease to confirm the topological nature of DNA binding. The input is shown in its covalently closed circular (CCC) form, as well as following linearization (L). Supernatant (S) and beads (B) fractions of the cohesin pulldown are shown from reactions in the presence or absence of the cohesin loader, with or without PstI digestion.

(C) Micrococcal nuclease digestion of the chromatinized plasmid used as a template in the cohesin loading reaction shown in Figure 3B, confirming efficient and even nucleosome assembly.

Figure S4

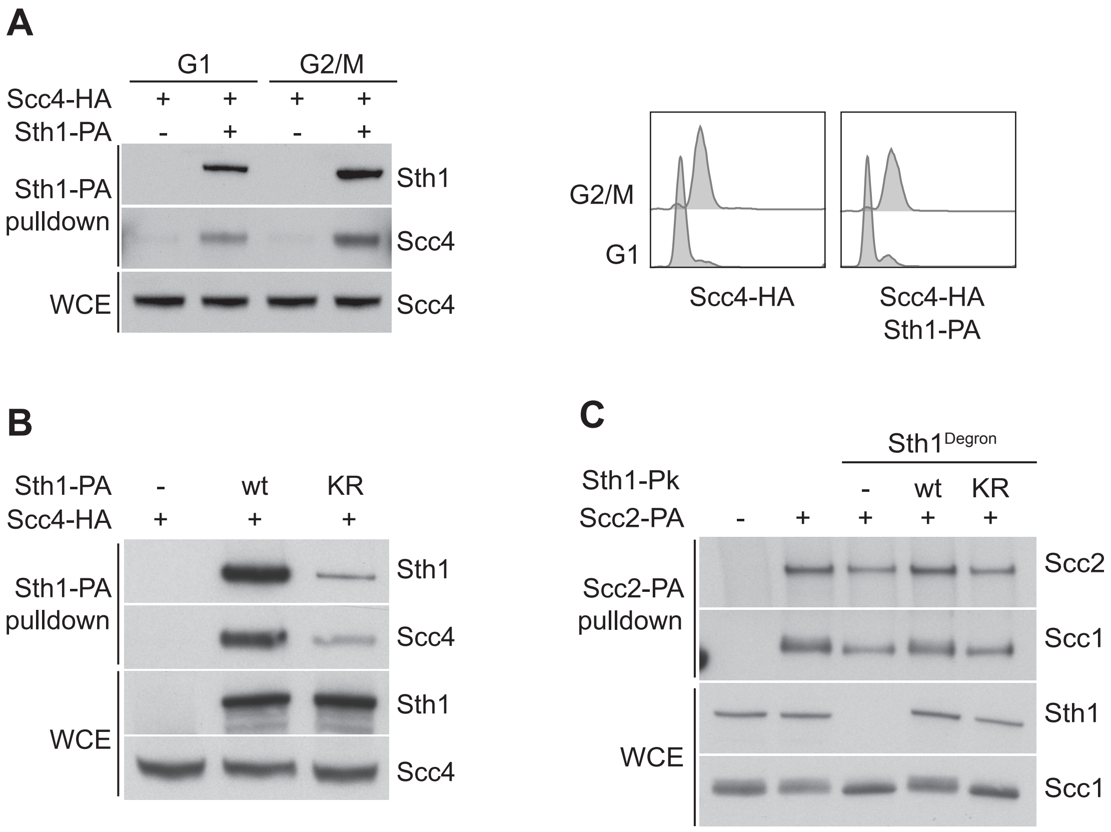
A
G1
G2/M
Scc4-HA
Sth1-PA
Sth1-PA pulldown
WCE
Sth1
Scc4
Scc4
G2/M
G1
Scc4-HA
Scc4-HA Sth1-PA
B
Sth1-PA
Scc4-HA
wt
KR
Sth1-PA pulldown
WCE
Sth1
Scc4
Sth1
Scc4
C
Sth1Degron
Sth1-Pk
Scc2-PA
wt
KR
Scc2-PA pulldown
WCE
Scc2
Scc1
Sth1
Scc1

Figure S4. Additional Interaction Analyses between RSC, Cohesin Loader and Cohesin, Related to Figure 4

(A) RSC interaction with the cohesin loader in G1 and mitosis. As in Figure 4A, but cell extracts were prepared from aliquots of a culture arrested in G1, or following release into nocodazole-imposed mitotic arrest. FACS analysis of DNA content is shown to confirm cell cycle synchrony.

(B) $Sth1^{K501R}$ interacts with the cohesin loader. Cell extracts were prepared, following depletion of endogenous Sth1, from $Sth1^{Degron}$ strains that expressed protein A-tagged wild type Sth1 or $Sth1^{K501R}$. Sth1 was adsorbed to IgG beads and coprecipitation of Scc4 was analyzed by immunoblotting. Despite similar expression levels, protein A pulldown of $Sth1^{K501R}$ was reproducibly less efficient compared to wild type Sth1. We do not know the reason for this difference. Based on the respective levels of Sth1 or $Sth1^{K501R}$ pulldown, coprecipitation of Scc4 appeared equally efficient.

(C) RSC-independent interaction between cohesin and the cohesin loader. Cell extracts were prepared from the indicated strains following Sth1 depletion, or from the indicated control strains. Protein A-tagged Scc2 was precipitated and coprecipitation of Scc1 was analyzed by immunoblotting.

Figure S5

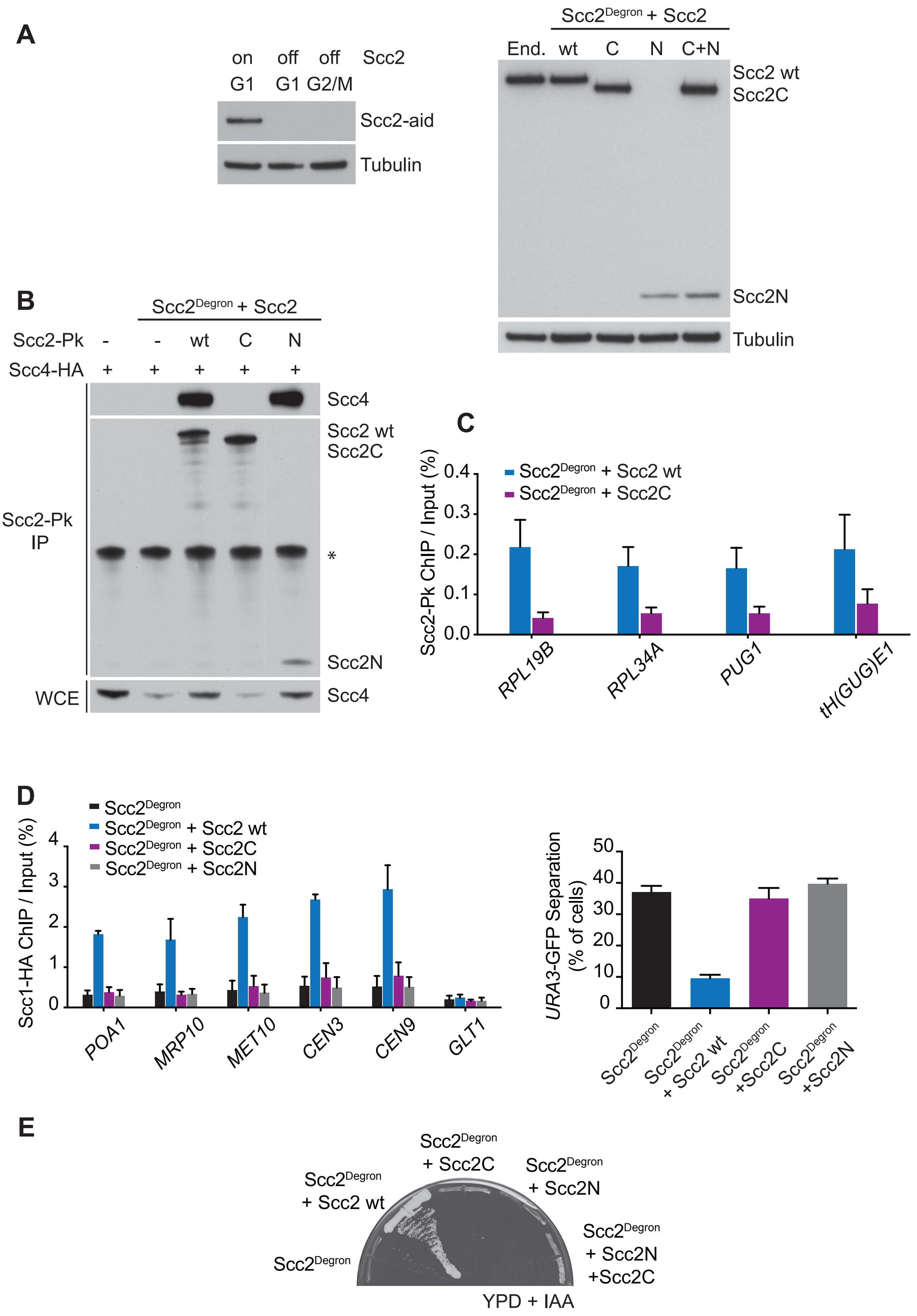
A
on G1
off G1
off G2/M
Scc2
Scc2-aid
Tubulin
Scc2Degron + Scc2
End.
wt
C
N
C+N
Scc2 wt
Scc2C
Scc2N
Tubulin
B
Scc2Degron + Scc2
Scc2-Pk - - wt C N
Scc4-HA + + + + +
Scc4
Scc2 wt
Scc2C
Scc2-Pk IP
*
Scc2N
WCE
Scc4
C
Scc2-Pk ChIP / Input (%)
Scc2Degron + Scc2 wt
Scc2Degron + Scc2C
RPL19B
RPL34A
PUG1
tH(GUG)E1
D
Scc2Degron
Scc2Degron + Scc2 wt
Scc2Degron + Scc2C
Scc2Degron + Scc2N
Scc1-HA ChIP / Input (%)
POA1
MRP10
MET10
CEN3
CEN9
GLT1
URA3-GFP Separation (% of cells)
Scc2Degron
Scc2Degron + Scc2 wt
Scc2Degron +Scc2C
Scc2Degron +Scc2N
E
Scc2Degron + Scc2C
Scc2Degron + Scc2N
Scc2Degron + Scc2 wt
Scc2Degron + Scc2N +Scc2C
Scc2Degron
YPD + IAA

Figure S5. Additional Characterization of the Cohesin Loader Modules, Related to Figure 5

(A) Endogenous Scc2 levels before and after depletion during G1 arrest and release into nocodazole-imposed G2/M arrest were monitored by immunoblotting with an anti-aid tag antibody (left). Immunoblot analysis of the various Scc2 fragments, fused to a Pk epitope tag, expressed under control of the Scc2 promoter at its endogenous or from an ectopic locus (right).

(B) Scc2N, but not Scc2C, interacts with and stabilizes Scc4. Cell extracts of the indicated strains were prepared after G1 arrest, endogenous Scc2 depletion and release into nocodazole-imposed mitotic arrest. The different Pk epitope-tagged Scc2 fragments were precipitated and copurification of Scc4 was analyzed by immunoblotting.

(C) Scc2C is undetectable at cohesin loading sites. Wild type Scc2 and Scc2C levels, following endogenous Scc2 depletion as in (A), were compared at four cohesin loading sites by ChIP followed by quantitative real-time PCR. Means and SEM of five independent experiments are shown. $Scc2^{Degron}$ + Scc2C $p < 0.01$; two-way ANOVA test compared to $Scc2^{Degron}$ + Scc2 wt.

(D) Neither Scc2C nor Scc4-Scc2N suffices for cohesin loading. Endogenous Scc2 was depleted in G1 before cells expressing the indicated Scc2 variants were released into nocodazole-imposed mitotic arrest. Cohesin levels at five cohesin binding sites and a negative control site were analyzed by ChIP followed by quantitative real-time PCR. Means and SEM of three independent experiments are shown. $Scc2^{Degron}$ + Scc2 wt $p < 0.01$; $Scc2^{Degron}$ + Scc2C and $Scc2^{Degron}$ + Scc2N p not significant; two-way ANOVA test compared to $Scc2^{Degron}$. Sister chromatid cohesion at the GFP-marked *URA3* locus was scored in a similar experiment. The means and SEM of three independent experiments are shown. $Scc2^{Degron}$ + Scc2 wt $p < 0.01$; $Scc2^{Degron}$ + Scc2C and $Scc2^{Degron}$ + Scc2N p not significant; Student's t-test compared to $Scc2^{Degron}$.

(E) Scc4-Scc2N and Scc2C must be linked to support cell growth. The ability of the respective Scc2 variants to restore viability following $Scc2^{Degron}$ depletion was tested by streaking the indicated strains on rich YPD medium containing methionine, supplemented with indole-3-acetic acid (IAA).

Figure S6

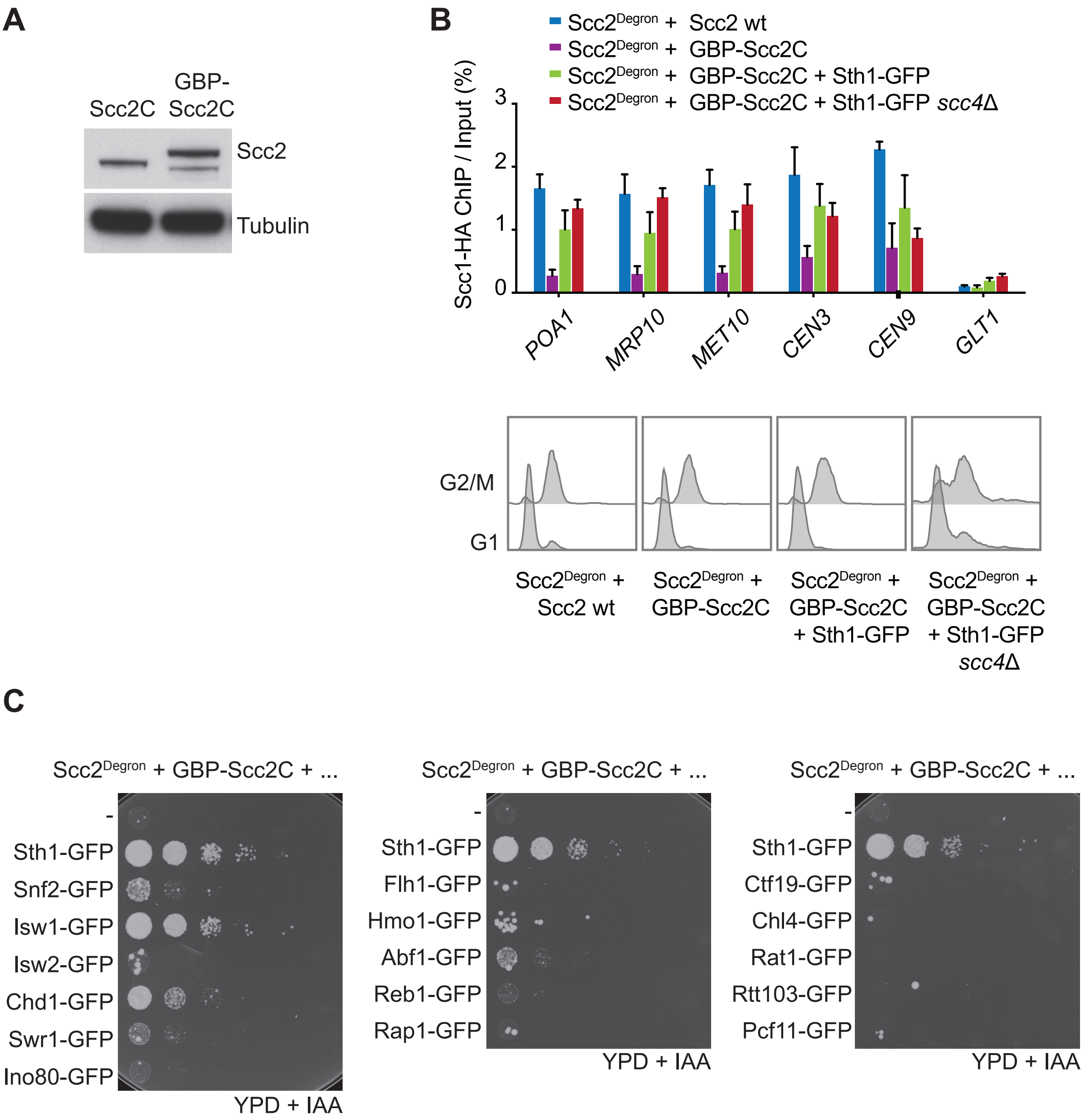
A
Scc2C
GBP-Scc2C
Scc2
Tubulin
B
Scc2Degron + Scc2 wt
Scc2Degron + GBP-Scc2C
Scc2Degron + GBP-Scc2C + Sth1-GFP
Scc2Degron + GBP-Scc2C + Sth1-GFP scc4Δ
Scc1-HA ChIP / Input (%)
3
2
1
0
POA1
MRP10
MET10
CEN3
CEN9
GLT1
G2/M
G1
Scc2Degron + Scc2 wt
Scc2Degron + GBP-Scc2C
Scc2Degron + GBP-Scc2C + Sth1-GFP
Scc2Degron + GBP-Scc2C + Sth1-GFP scc4Δ
C
Scc2Degron + GBP-Scc2C + ...
-
Sth1-GFP
Snf2-GFP
Isw1-GFP
Isw2-GFP
Chd1-GFP
Swr1-GFP
Ino80-GFP
YPD + IAA
Scc2Degron + GBP-Scc2C + ...
-
Sth1-GFP
Flh1-GFP
Hmo1-GFP
Abf1-GFP
Reb1-GFP
Rap1-GFP
YPD + IAA
Scc2Degron + GBP-Scc2C + ...
-
Sth1-GFP
Ctf19-GFP
Chl4-GFP
Rat1-GFP
Rtt103-GFP
Pcf11-GFP
YPD + IAA

Figure S6. Additional Data to Characterize Engineered Cohesin Loading by GBP-Scc2C, Related to Figure 6

(A) Expression of GBP-Scc2C, next to Scc2C, was analyzed by immunoblotting against the C-terminal Pk epitope tags contained on both proteins. The faster migrating GBP-Scc2C band is likely the product of proteolytic cleavage at a flexible linker sequence between the GBP tag and Scc2C, that might be sensitive to proteases.

(B) Scc4 is dispensable for cohesin loading by GBP-Scc2C in conjunction with Sth1-GFP. Cohesin loading onto chromosomes was assessed as in Figure 6C, but including a strain lacking Scc4. Means and SEM of three independent experiments are shown. $Scc2^{Degron}$ + Scc2 wt, $Scc2^{Degron}$ + GBP-Scc2C + Sth1-GFP and $Scc2^{Degron}$ + GBP-Scc2C + Sth1-GFP *scc4Δ* $p < 0.01$; two-way ANOVA test compared to the $Scc2^{Degron}$ + GBP-Scc2C strain. FACS analysis of DNA content confirmed the cell cycle stage at the time of endogenous Scc2 depletion and the mitotic arrest when the ChIP samples were taken. Note the reduced synchrony of cells lacking Scc4, suggesting that the chronic absence of Scc4 compromises cell proliferation.

(C) Cell growth as supported by GBP-Scc2C in conjunction with various putative chromatin receptor-GFP fusions. 10-fold serial dilutions of the indicated strains were spotted on YPD + IAA medium to deplete endogenous Scc2.

Table S2. **qPCR primers used in this study**

Name	Sequence (5' -3')
POA1-F	AAACGGCCACATCAAATACC
POA1-R	TCCAAGGGACTCCGAATATG
MRP10-F	ACCCCCTCTTCCCAGACTAA
MRP10-R	CCAGCACATTTAGGGCTCAT
MET10-F	ACTTGTGTGGCCCTACTTGG
MET10-R	CGACTTTGATGCCTCTTTCC
CEN3-F	CGCCACTTTAACAAATGTGC
CEN3-R	GCAGAACCACCGTAGCAGTT
CEN9-F	TGTCACCTGGCTGTTTTGAG
CEN9-R	TGGGTAATGTCAGCTGTGGA
GLT1-F	TTTGACCCCAGCACATGTTA
GLT1-F	GGGTGTGGAGTTTGTGGTCT
RPL23B-F	CCGTCAAGCTAAGTCTTGGAGAAG
RPL23B-R	CCTTAGGATTAGCGATGACACCAG
RPL34A-F	GTCTTGTGGGTCTTGGAAACAG
RPL34A-R	CAAAGTGTGGTGACTGTGGTAG
PUG1-F	GCGGCAAGCTCATCCTAAAT
PUG1-R	CCCACAATTGATTCGGTAGG
tH(GUG)E1-F	GAAACCCTGGTTCGATTCTAGGAG
tH(GUG)E1-R	GCTCTCATGATCACCACATCTGAC
CIN8-F	AGGGCACAACTAGATAAACAGCA
CIN8-R	GGGCCATTTGCATTACCTCAGTCA

Table S3. **Plasmids used in this study**

Name	Description	Purpose	Source
pSM15	YIplac204-Sth1-PK_3	Integration of wild type Sth1-PK_3 at *TRP1* locus	This study
pSM16	YIplac204-Sth1K^{501R}-PK_3	Integration of wild type $Sth1^{K501R}$-PK_3 at *TRP1* locus	This study
pSM32	YIplac204-Sth1-HA_6	Integration of wild type Sth1-HA_6 at *TRP1* locus	This study
pSM33	YIplac204-Sth1K^{501R}-HA_6	Integration of wild type $Sth1^{K501R}$-HA_6 at *TRP1* locus	This study
pSM51	YIplac204-Sth1-PA_2	Integration of wild type Sth1-PA_2 at *TRP1* locus	This study
pSM52	YIplac204-Sth1K^{501R}-PA_2	Integration of wild type $Sth1^{K501R}$-PA_2 at *TRP1* locus	This study
pCCD51	pBS-KSII-cut2-ARS1	Substrate for the cohesin loading assay	This study
	pCDFduet.H2A-H2B	Histone purification	Kurat *et al.*, 2017
	pETduet.H3-H4	Histone purification	Kurat *et al.*, 2017
pCFK1	pGEX-Nap1	Nap1 purification	Kurat *et al.*, 2017
pSM2	pRS303-Scc2-PK_3	Integration of wild type Scc2-PK_3 at *HIS3* locus	This study
pSM4	pRS303-Scc2-C-PK_3	Integration of Scc2-C-PK_3 at *HIS3* locus	This study
pSM8	pRS303-Scc2-N-PK_3	Integration of Scc2-N-PK_3 at *HIS3* locus	This study
pSM11	pRS304-Scc2-N-PK_3	Integration of Scc2-N-PK_3 at *TRP1* locus	This study
pSM30	pRS303-Scc2-HA_6	Integration of wild type Scc2-HA_6 at *HIS3* locus	This study
pSM31	pRS303-Scc2-C-HA_6	Integration of Scc2-C-HA_6 at *HIS3* locus	This study
pSM34	pRS303-Scc2-N-HA_6	Integration of Scc2-N-HA_6 at *HIS3* locus	This study
pSM50	pRS303-GBP-Scc2-C-PK_3	Integration of Scc2-GBP-C-PK_3 at *HIS3* locus	This study
